# Determination of macro-scale soil properties from pore scale structures: image-based modelling of poroelastic structures

**DOI:** 10.1098/rspa.2017.0745

**Published:** 2018-07-11

**Authors:** K. R. Daly, S. D. Keyes, T. Roose

**Affiliations:** School of Engineering Science, University of Southampton, Southampton SO17 1BJ, England, UK

**Keywords:** poroelasticity, multiscale analysis, homogenization, soil science, image-basedmodelling

## Abstract

We show how a combination of X-ray computed tomography (X-CT) and image-based modelling can be used to calculate the effect of moisture content and compaction on the macroscopic structural properties of soil. Our method is based on the equations derived in Daly & Roose (2018 *Proc. R. Soc. A* **474**, 20170141. (doi:10.1098/rspa.2017.0141)), which we have extended so they can be directly applied to the segmented images obtained from X-CT. We assume that the soils are composed of air-filled pore space, solid mineral grains and a mixed phase composed of both clay particles and water. We considered three different initial soil treatments, composed of two different compaction levels and two different moisture contents. We found that the effective properties of the soils were unaffected by compaction over the range tested in this paper. However, changing the moisture content significantly altered the hydraulic and mechanical properties of the soils. A key strength of this method is that it enables the optimization or even design of soils composed from different constituents, with specific mechanical and hydraulic properties.

## 1. Introduction

The mechanical and structural properties of soil determine its ability to withstand compression, which is recognized as a serious threat to soil health [1]. Soil compaction due to heavy machinery is known to adversely affect soil hydraulic properties and can have effects which last for timescales greater than 15 years [2]. There are numerous analytic studies which model the effects of soil compaction, see [3,4] and references therein. However, while these models provide some insight, there are still numerous challenges associated with their use, particularly in heterogeneous soils [3]. A key challenge in understanding soil mechanical properties is that soils are inherently multiscale, with macroscopic poro-elastic properties that are dependent on the precise details of the microstructure [5]. Linking this micro-scale modelling to the macro-scale is an important challenge [6]. Recently, there has been an increase in the use of different imaging and image-based methods to study the deformation of soils. On the micro-scale, imaging techniques are often used to aid understanding of the observable macro-scale deformation [7–10]. These techniques include X-ray computed tomography (X-CT) combined with digital volume correlation [8,11], particle image velocimity [9] and X-CT coupled with flow modelling [10]. In addition, micro-model fabrication and imaging can be used to study the flow of fluids in porous media [12].

From a mathematical modelling perspective, one way to describe the mechanics of soils is through the constitutive relations of Biot [13–15]. These equations are widely used in soils [16,17] and are based on the idea that stress, strain, pressure and the change in fluid fraction can be linearly related. It has been shown that the theory of Biot can be derived using homogenization for both small and large deformations [18,19]. Homogenization theory is a mathematical technique that allows macro-scale equations to be derived based on a set of representative equations, known as cell problems, which are solved on an underlying periodic microstructure [20,21].

Homogenization has been widely used in the field of porous media [20]. Typically, it is used to describe saturated fluid flow [22,23], two-phase flow [24], diffusion [25,26] and poroelasticity [27]. In addition to hierarchical homogenization, in which multiple levels of homogenization are applied, has been used to describe fluid flow [28] and poroelasticity [29]. Typically, poroelastic homogenization studies focus on two different interacting phases such as elastic solids and fluids [18,19], poroelastic materials and fluids [29] or viscoelastic materials and fluids [27]. In addition, partially saturated porous media have been analysed using thermodynamic mixture theory to link soil water retention and effective stress [30], this approach does not provide the direct multi-scale link between observed behaviours and microstructural details, which is captured using homogenization.

**Figure 1.**
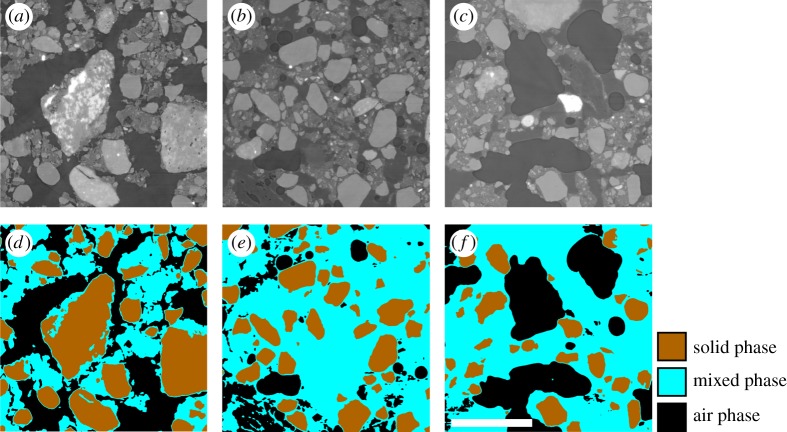
Different segmented soil samples used and segmentation used for modelling. Left to right shows different soil samples and top to bottom shows different phases of segmentation. (*a*,*d*) Drier compact soil, (*b*,*e*) wet compact, (*c*,*f*) wet non-compacted. The top set of images (*a*–*c*) show the original grey scale images obtained via X-CT. The bottom row of images (*d*–*f*) show the segmentation of the soil into three phases (scale bar, 1 mm). (Online version in colour.)

**Figure 2.**
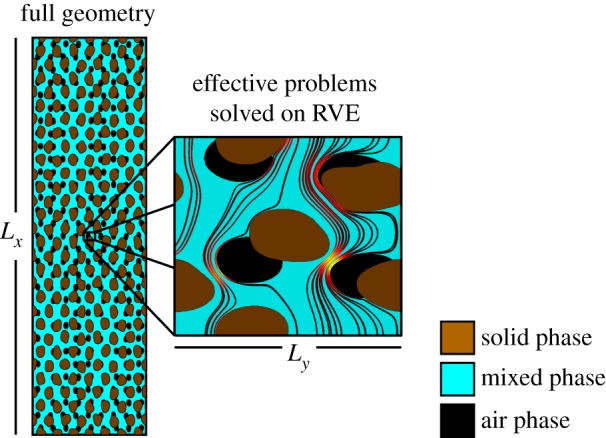
Schematic of model developed in [31]. Rather than solve a representative set of equations on a full geometry of size *L*_*x*_ (left), a set of mathematically derived cell problems are solved on a representative volume element of size *L*_*y*_. The cell problems are computationally tractable and feed into a geometry independent model through a set of effective parameters. Illustrated in this geometry are the three phases considered within the model and representative Darcy flow streamlines through the mixed phase, warmer colours correspond to a greater Darcy flux of water. (Online version in colour.)

In a recent paper, [31], we used homogenization theory to derive an averaged macro-scale model for the poroelastic properties of soil composed of three distinct phases: solid grains, air-filled pore space and a mixed phase comprised of small clay particles and water. This is illustrated in figure 1. We considered the mixed phase as a poroelastic mixture composed of elastic colloids and pore water [13,32]. Within the soil, the solid particles move through rigid body translations, and the air-filled pore space acts as an incompressible viscous fluid. Starting from a full set of equations, which described this set of physics, we used homogenization to derive a set of approximate (but computationally tractable) macroscopic equations for the case of large structural deformation [19,21]. The resulting equations were parametrized through a set of cell problems solved on a representative geometry (figure 2). These equations were validated by comparing the results predicted by this method to those calculated by solving the full set of equations for an idealized geometry. In this paper, we will use these equations, [31], in combination with image-based modelling to study the macroscopic properties of a sandy loam, the most common soil type in the UK. Image-based modelling refers to the solution of equations on geometries obtained directly from X-CT. This technique has been widely used in the porous media literature [33,34] and, in the context of soils has been used to study saturated flow properties [10,35], diffusion [36] and to capture partially saturated soil properties [37]. Here, we use image-based modelling to compare the structural properties of soils subject to different initial conditions. Specifically, we consider soil subject to two different levels of compaction and two different water contents. The novel contribution to this work lies firstly in the derivation of symmetry reduced cell problems from the model presented in [31]. These cell problems can be applied directly to complex image-based geometries. The second contribution is the direct application of these techniques to three different soil treatments in order to compare and contrast different soils and enable the potential design and optimization of soil structures with specific mechanical and hydraulic properties.

We show that the image-based modelling method can determine distinct differences between the properties of these soils and that it can be used to link the microscopic structural properties of soils to the observable macroscopic properties. We found that the effective properties of the soils were unaffected by the initial compaction. However, changing the initial moisture content significantly altered the hydraulic and mechanical properties of the soils. We note that the method employed here does not determine the absolute value of macro-scale properties such as the effective mobility, stiffness, pressure induced stress or increment of fluid content due to pressure or dilation. Rather, it determines the link between what is observed on each scale. This is due to the unknown micro-scale physical properties of the mixed phase, namely the drained Poisson ratio, the shear modulus and the permeability of the mixed phase. We note that, while the focus of this paper is the effective properties of soil, the mathematics could be applied to any composite material composed of solid, poroelastic, elastic or viscous constituents.

## 2. Image preparation

To understand the effect of moisture and consolidation on soil poroelastic properties, we considered six replicates of three different soil conditions, 18 samples in total. A full description of how each condition was prepared is given in [11]. In brief, the soil was a sand-textured Eutric Cambisol collected from a surface plot at Abergwyngregyn, North Wales. The soil was initially sieved to less than 5 mm and air-dried for 2 days at 23 ± 1°C before being sieved again to between 1.18 and 0.6 mm. The three soil treatments were: a loose textured soil with a high water content, a mechanically consolidated soil with a high water content and a mechanically consolidated soil with a low water content.

To produce samples for the wet loose treatment dry soil was introduced to the working volume and was lightly consolidated via tapping [38] to a volume of 0.3 ml. The soil was hydrated to a water content of 25% b.v. by adding 0.075 ml of water via pipette. To produce samples for the wet consolidated treatment, dry soil was introduced as before, to a volume of 0.2 ml, following which 0.0375 ml of water was added via pipette. Another 0.2 ml of soil was introduced, followed by another 0.0375 ml of water. The resulting 0.4 ml working volume was then compacted to a volume of 0.3 ml using a plastic ram, giving a water content of 25% b.v. To produce samples for the dry consolidated treatment, a 3 mm depth of dry soil was layered between two moistened 185 mm diameter filter papers (Whatman No. 2), which were sealed between sheets of polythene film, and allowed to equilibrate for 5 h at 21 ± 1°C. The soil layer was gently homogenized by pouring into a beaker and re-scattering at 0.5 h intervals. This produced a medium with a gravimetrically determined bulk volumetric water content of 11.2% b.v. This soil was introduced to the working volume as before, consolidating lightly via tapping to a volume of 0.4 ml, following which the 0.4 ml working volume was compacted to a volume of 0.3 ml via a plastic ram, giving a water content of 14.9% b.v.

Tomographic data were acquired at the TOMCAT beamline of the Swiss Light Source (Villigen, Switzerland), using a 19 kV monochromatic beam condition. For each tomogram, 750 projections were continuously acquired at a 75 ms exposure, with 30 dark- and 50 flat-field images acquired for radiograph correction. Each tomographic acquisition took approximately 60 s in total. The attenuated beam was scintillated by a LuAG:Ce film of 20 μm thickness, and resultant images digitized using a PCO Edge 5.5 camera. This set-up provided a total of 4 × magnification, yielding a pixel size of 1.6 μm. A sample-to-detector distance of 50 mm provided an intermediate degree of propagation phase contrast.

An in-house implementation of the single-distance phase retrieval algorithm, [39], was applied to all projections, which were then reconstructed to 16-bit volumes via filtered back projection, using a computing cluster and an implementation, [40], of the Gridrec algorithm [41]. This produced SRXCT volumes with a voxel edge length of 1.6 μm. The soil phases were separated using a global histogram method to produce a three-phase image consisting of solid phase, mixed phase and air-filled pore space. To separate touching particles, a watershed algorithm was applied [42]. Figure 1 shows the original grey scale image, and the initial three-phase segmentation of the imaged geometry.

## 3. The averaged model

In our previous paper [31], we used the method of homogenization to derive an averaged poroelastic model for soil deformation. As we will use this model to relate the data obtained from X-CT to the macroscopic properties of the soils, we briefly summarize the method, which is divided into three steps. Further details of the model are provided in electronic supplementary material, S1. The first step, electronic supplementary material, SS1(a), is to form a complete micro-scale description of the phases involved. These equations are based on underlying physics and could, in principle, be solved on their own to study the poroelastic properties of soil. These equations assume that the imaged soil is in equilibrium (something which is essential in obtaining high quality X-CT images). Hence, the saturation is fixed and the air-mixed phase interface is known *a priori*.

**Figure 3.**
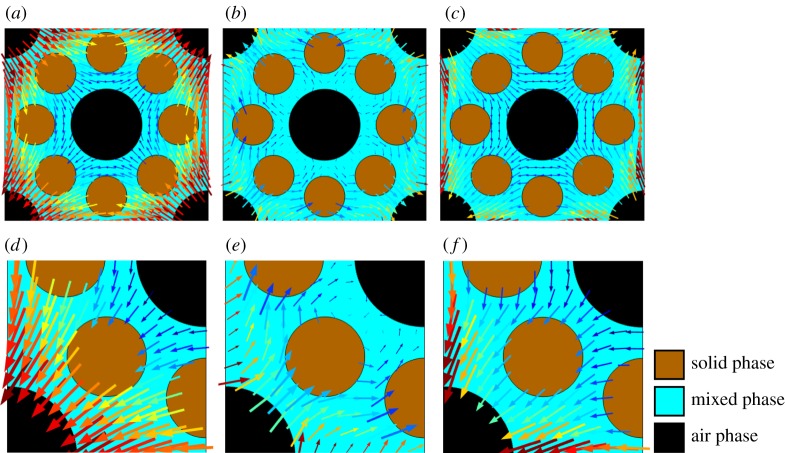
Schematic showing the shear deformation of soil induced by a macro-scale strain gradient. The arrows indicate the displacement direction and relative magnitude with red corresponding to larger displacement and blue corresponding to smaller displacement. The top row (*a*–*c*) shows the displacement on a single periodic unit for (*a*) the macro-scale displacement, (*b*) the micro-scale strain gradient, i.e. the solution to the cell problem with periodic boundary conditions and (*c*) the total displacement, i.e. the sum of (*a*) and (*b*). The bottom row (*d*–*f*) shows the equivalent problem on $\frac{1}{4}$ of the geometry, i.e. the symmetry reduced problem. (*d*) Shows the macro-scale displacement, (*e*) the micro-scale displacement, i.e. the solution to the symmetric cell problem and (*e*) the total displacement, i.e. the sum of (*d*) and (*e*). (Online version in colour.)

Solving these equations on images obtained from X-CT is computationally prohibitive. Hence, we derived an approximation to these equations [31]. The aim was to derive a set of averaged equations that do not explicitly consider each detail of the underlying soil geometry. However, the parameters that appear in these equations are derived through a series of ‘cell problems’, which capture the underlying geometry through a representative (but small) soil geometry, electronic supplementary material, SS1(b). Physically, we can understand this process as a series of perturbations. Given an initial geometry (obtained from X-CT or otherwise) and a complete micro-scale set of physics, we calculate the effect of applying pressure and displacement gradients. These gradients, which we term the macro-scale gradients, are small with respect to the size of the representative geometry and, importantly, are independent of the precise geometrical features. This last assumption means that it is impossible for the resulting pressure and displacement fields to satisfy the micro-scale equations. Hence, we introduce a set of micro-scale fields for displacement and pressure driven by the macro-scale fields. This is illustrated in figure 3.

Finally, electronic supplementary material, SS1(c), an average is performed that takes into account the micro- and macro-scale pressure and displacement gradients. The result is an averaged set of equations, which captures the poroelastic properties of the soils on the macro-scale. These equations are parametrized by the micro-scale geometry and physical properties.

### (a) The micro-scale equations

The first stage of the homogenizaiton procedure was to consider a microscopic description of the soil physical properties. We considered the soil domain to be composed of a solid phase *Ω*_s_, an air phase *Ω*_a_ and a mixed phase *Ω*_m_. The phase boundaries are defined as *Γ*_as, *j*_, *Γ*_ms, *j*_ and *Γ*_am_ for the air–solid, mixed–solid and air–mixed boundaries, respectively. A full list of parameters and their definitions are given in table 1. The mixed phase is composed of water and smaller mineral phases and is mathematically described as a poroelastic mixture [13–15,32]. We make no assumptions about the volume ratio of water to smaller mineral phases within the mixed phase. In reality, the mixed phase of a drier soil would have a much higher fraction of solid than water, while a wetter soil would have a much larger fraction of liquid than solid. We also define ${\hat{n}}^{\mathrm{am}}$ as the unit normal vector on the air–mixed phase interface pointing into the mixed phase domain, ${\hat{n}}^{\mathrm{as}}$ as the unit normal vector on the air–solid interface pointing into the solid domain, and ${\hat{n}}^{\mathrm{ms}}$ as the unit normal vector on the mixed phase–solid interface pointing into the solid domain.

**Table 1.** Parameters and variables used in the macro-scale model, the different phases are denoted through the variable *i* = {*a*, *m*, *s*, *w*}.

variable	description	definition
*p*^*i*^	pressure in the phase *i*	solution
± *bu*^*i*^	displacement in the phase *i*	solution
*ρ*^*i*^	density of phase *i*	literature
*μ*^*i*^	viscosity of phase *i*	literature
*g*	gravitational acceleration	literature
*L*_*x*_	macro-scale length scale	literature
*L*_*y*_	micro-scale length scale	X-CT geometry
*k*	water permeability	literature
*ν*	Poisson ratio of mixed phase	literature
*G*	shear modulus of mixed phase	literature
${\hat{n\;n}}^{ij}$	surface normal pointing from domain *i* to domain *j*	X-CT geometry
*Ω*_*i*_	domain corresponding to phase	X-CT geometry
*Γ*_*ij*_	boundary between phase *i* and phase *j*	X-CT geometry
*δ*_1_	water–air mobility ratio	(3.1)
*δ*_2_	density of mixed phase relative to water	(3.1)
*δ*_3_	density of air phase relative to water	(3.1)
*δ*_4_	density of solid phase relative to water	(3.1)
*g*^w^	dimensionless gravity	(3.1)
*p*^c^	dimensionless capillary pressure	(3.1)
∥*Ω*_m_∥	volume fraction of mixed phase	(3.7)
∥*Ω*_a_∥	volume fraction of air phase	(3.7)
± *bζ*_*k*_	local air velocity coefficient	(3.2)
*ω*^a^_*k*_	local air pressure coefficient	(3.2)
*ω*^m^_*k*_	local mixed phase pressure coefficient	(3.3)
± *bκ*^u^_*pq*_	local mixed phase displacement coefficient	(3.4)
${\hat{\kappa \;\kappa }}_{pq}^{u}$	local solid phase displacement coefficient	(3.4)
± *bκ*^p^	local mixed phase pressure coefficient	(3.5)
${\hat{\kappa \;\kappa }}^{p}$	local solid phase pressure coefficient	(3.5)
*C*^u^_*ijkl*_	effective stiffness tensor	(3.6*a*)
*C*^p^_*ij*_	pressure–induced strain coefficient	(3.6*b*)
*g*^eff^	effective gravity	(3.6*c*)
*K*^w^_*ij*_	effective water mobility	(3.8*a*)
*A*^u^_*ij*_	increment in water content due to changes in strain	(3.8*b*)
*A*^p^	increment in water content due to change sin pressure	(3.8*c*)
*K*^a^_*ij*_	effective air mobility	(3.9*a*)
*B*^u^_*ij*_	increment in air content due to changes in strain	(3.9*b*)
*B*^p^	increment in air content due to changes in pressure	(3.9*c*)

The three-phase poroelastic material is described in terms of solid displacements, and fluid pressures and velocities. The effective equations are derived and calculated based on the non-dimensional constants

3.1 $$\delta_{1}=\frac{\mu^{a}k}{\mu^{w}L_{y}^{2}},\;\delta_{2}=\frac{\rho^{m}}{\rho^{w}},\;\delta_{3}=\frac{\rho^{a}}{\rho^{w}},\;\delta_{4}=\frac{\rho^{s}}{\rho^{w}},\;g^{w}=\frac{\rho^{w}gL_{x}^{2}}{GL_{y}}\;\mathrm{and}\;p^{c}=\frac{\gamma \kappa L_{x}}{GL_{y}},$$

where *ρ*^w^ is the water density, *ρ*^m^ is the density of the solid part of the mixed phase, *g* is the acceleration due to gravity, *κ* is the mean curvature of the air–water interface and *γ* is the surface tension. To confirm our assumption, that the soils are in equilibrium, we can estimate the capillary number (on the micro-scale) as *Ca* = (*kG*)/(*L*_*y*_*γ*) ≈ 1.38 × 10^3^, assuming *k* = 10^−10^ m^2^, *G* = 10^8^ Pa, *L*_*y*_ = 10^−3^ m and *γ* = 0.72 × 10^−3^ Pa m. Hence, we note that capillary forces dominate over viscous forces and the dynamic motion of the interface occurs on a timescale much quicker than the one considered in this paper. We note this estimate of *Ca* is likely to be an overestimate as the length scale used is that of the representative volume rather than the actual pore size (which will be smaller).

### (b) Cell problems

To derive an averaged model for the poroelastic behaviour of soils, the method of homogenization was applied [31]. The approach involved deriving a set of cell problems which capture the effect of the underlying geometry on the mechanical and fluid properties of the soil. Here, our approach differs slightly from the one described in [31]. The key assumption used in [31] was that the geometry is periodic, i.e. it is composed of regularly repeating units. In reality, soils will not have a perfectly periodic structure. Hence, we follow the approach of enforcing periodicity via reflection of the imaged geometry [35].

This symmetry reduction allows us to overcome the lack of periodicity in the imaged soils [35,43]. However, it comes at the price of forcing us to make assumptions regarding the structure and anisotropy of the imaged geometry. Clearly the soil image (figure 1) is not composed of regularly repeating units. Hence, enforcing periodicity changes the soil structure. However, assuming that the soil structure is sufficiently homogeneous, enforcing periodicity by reflection will only create a significantly altered soil structure on the boundaries of the geometry. As the volume considered is increased, the total volume fraction that is altered by the reflection is reduced. Hence, by increasing the volume of soil considered, we would expect the effective properties measured to converge to those of the soil sample.

From a physical perspective, a periodic geometry composed of arbitrary repeating units could be isotropic, anisotropic or even chiral. However, if we assume that the representative unit has reflective symmetry in the three principal Cartesian directions, then we cannot construct a chiral material. In addition, the only anisotropic materials which respect these symmetries are those which are already aligned along the principal coordinate axes about which we make our reflections. In other words, we are only able to detect the diagonal components of any anisotropic material. These assumptions are necessary in order to obtain an appropriate representation of the soil on which the cell problems can be solved. In addition, we have no reason to believe that these soil samples will have a chiral structure or that they will exhibit any off-axes anisotropy. It is possible that, as gravity acts on the soil in a single direction (aligned with the coordinate axes), there will be some variation between the effective parameters in the *y*_1_, *y*_2_ and *y*_3_ directions. The symmetry reduction we have considered will allow us to detect this difference.

The cell problems derived in [31] describe the Darcy flow of air through the porespace, the impeded Darcy flow of water in the mixed phase, the local stiffness, and the pressure-induced stress. Our aim is to obtain a reflected cell problem based on the reflections *y*_*k*_ →  − *y*_*k*_ for *k* = {1, 2, 3}. The reflected cell problems are identical to the original cell problems under an appropriate translation of the dependent variable, (*u* →  − *u* for example). By finding these translations, we can infer that if the system is invariant under the translation *y*_*k*_ →  − *y*_*k*_ and *u* →  − *u*, then *u* must be odd about the plane of reflection. Hence, an appropriate boundary condition would be *u* = 0. By repeating this for all directions, we can infer a set of boundary conditions to impose on the edges of our domain. Using the symmetry reduction techniques (detailed in electronic supplementary material, S1) we find the cell problem

3.2*a* $$\begin{array}{rl} & \nabla_{y}^{2}\zeta_{k}-\nabla_{y}\omega_{k}^{a}={\hat{e}}_{k},\;y\in \Omega_{a}, \end{array}$$

3.2*b* $$\begin{array}{rl} & \nabla_{y}\cdot \zeta_{k}=0,\;y\in \Omega_{a} \end{array}$$

3.2*c* $$\begin{array}{rl} \;\text{and}\; & \zeta_{k}=0,\;y\in \Gamma_{\mathrm{am}}\cup \Gamma_{\mathrm{as}}, \end{array}$$

where periodic boundary conditions were assumed. For the imaged geometries, we assume that the imaged geometry comprises 1/8th of the representative volume of soil. The total geometry can be reconstructed by reflecting the imaged data in *y*_1_ = 0, *y*_2_ = 0 and *y*_3_ = 0. By imposing the translations *y*_a_ →  − *y*_a_, for *a* = {1, 2, 3}, we derive the following symmetry boundary conditions:

3.2*d* $$\omega_{k}^{a}=0,\frac{\partial }{\partial y_{k}}({\hat{e}}_{k}\cdot \zeta_{k})=0,\;{\hat{e}}_{p}\cdot \zeta_{k}=0,\;p\neq k,\;y\in \partial y_{k}$$

and

3.2*e* $$\frac{\partial }{\partial y_{j}}\omega_{k}^{a}=0,\;\frac{\partial }{\partial y_{p}}({\hat{e}}_{p}\cdot \zeta_{k})=0,\;{\hat{e}}_{j}\cdot \zeta_{k}=0,\;p\neq j,\;j\neq k,\;y\in \partial y_{j},$$

where ***ζ***_*k*_ and *ω*^a^_*k*_ are the local velocity and pressure coefficients driven by a large-scale pressure gradient in the air phase. These can be determined by solving equations (3.2) assuming *Ω*_a_ is the 1/8th volume air domain. *Γ*_am_ and *Γ*_as_ are the 1/8th area solid and mixed phase boundaries, ∂***y***_*k*_ is the union of the boundaries at *y*_*k*_ = 0 and *y*_*k*_ = 1/2. The boundary conditions (3.2d) and (3.2e) enforce a normal flow condition in the direction of forcing and a slip condition in the non-forced directions.

Similarly the symmetric cell problem for the Darcy flow of water in the mixed phase is

3.3*a* $$\begin{array}{rl} & \nabla_{y}^{2}\omega_{k}^{m}=0,\;y\in \Omega_{m}, \end{array}$$

3.3*b* $$\begin{array}{rl} & {\hat{n}}^{\mathrm{ms}}\cdot \nabla_{y}\omega_{k}^{m}+{\hat{n}}^{\mathrm{ms}}\cdot {\hat{e}}_{k}=0,\;y\in \Gamma_{\mathrm{ms}} \end{array}$$

3.3*c* $$\begin{array}{rl} \;\text{and}\; & {\hat{n}}^{\mathrm{am}}\cdot \nabla_{y}\omega_{k}^{m}+{\hat{n}}^{\mathrm{am}}\cdot {\hat{e}}_{k}=0,\;y\in \Gamma_{\mathrm{am}}, \end{array}$$

with the symmetry boundary conditions

3.3*d* $$\omega_{k}^{m}=0,\;y\in \partial y_{k}$$

and

3.3*e* $$\frac{\partial }{\partial y_{j}}\omega_{k}^{m}=0,\;y\in \partial y_{j},$$

where *ω*^m^_*k*_ is the pressure coefficient driven by a large-scale pressure gradient in the mixed phase.

The symmetry boundary conditions for the cell problems, arising from the compressibility of the mixed phase, are more complex. From a macroscopic point of view, applying a strain to the soil sample will cause deformation of all components in the soil. However, as we have assumed that the soil minerals move as rigid bodies, they cannot be deformed. Hence, any macro-scale deformation must be coupled with a micro-scale variation in soil deformation which is equal and opposite to the macro-scale deformation on the solid grains (see [31] for a more formal derivation). This deformation is captured by solving

3.4*a* $$\begin{array}{rl} & \nabla_{y}\cdot \alpha_{pq}^{u}=0,\;y\in \Omega_{m}, \end{array}$$

3.4*b* $$\begin{array}{rl} & \alpha_{pq}=e_{y}(\kappa_{pq}^{u})+\frac{\nu }{1-2\nu }(\nabla_{y}\cdot \kappa_{pq}^{u})I,\;y\in \Omega_{m} \end{array}$$

3.4*c* $$\begin{array}{rl} \;\text{and}\; & {\hat{n}}^{\mathrm{am}}\cdot \left[\alpha_{pq}^{u}+\frac{{\hat{e}}_{p}{\hat{e}}_{q}+{\hat{e}}_{q}{\hat{e}}_{p}}{2}+\frac{{\hat{e}}_{p}\cdot {\hat{e}}_{q}}{2}\frac{\nu }{1-2\nu }I\right]=0,\;y\in \Gamma_{\mathrm{am}}, \end{array}$$

where ***κ***^u^_*pq*_ is the displacement coefficient driven by a large-scale strain gradient and *α*^u^_*pq*_ is the corresponding stress coefficient. The boundary conditions on the boundary ∂*y*_*k*_ can be written in the compact form

3.4*d* $${\hat{e}}_{k}\cdot [{\hat{e}}_{p}{\hat{e}}_{q}+{\hat{e}}_{q}{\hat{e}}_{p}+{\hat{e}}_{p}{\hat{e}}_{p}+{\hat{e}}_{q}{\hat{e}}_{q}-I]\cdot \kappa_{pq}^{u}=0,\;y\in \partial y_{k},$$

3.4*e* $${\hat{e}}_{k}\cdot [{\hat{e}}_{p}{\hat{e}}_{p}+{\hat{e}}_{q}{\hat{e}}_{q}]\alpha_{pq}^{u}[{\hat{e}}_{p}{\hat{e}}_{p}+{\hat{e}}_{q}{\hat{e}}_{q}]\cdot {\hat{e}}_{k}=0,\;p\neq q,\;y\in \partial y_{k}$$

or

3.4*f* $${\hat{e}}_{j}\cdot \alpha_{pq}^{u}\cdot {\hat{e}}_{j}=0,\;j\neq k,\;p=q,\;y\in \partial y_{k}.$$

These boundary conditions are illustrated for the case *p*≠*q* in figure 3. The boundary conditions on the solid grains are more complex and we consider the cases *p* = *q* and *p*≠*q* separately. For the case *p* = *q*, we observe that, for any solid grain that intersects a boundary, *y*_*k*_ = 0 or *y*_*k*_ = 0.5 for *k* = {1, 2, 3}, the net displacement of that grain in the direction ${\hat{e}}_{k}$ must be zero. In addition, we find that the stress condition projected into the direction ${\hat{e}}_{k}$ is automatically satisfied. Therefore, for a particle that intersects the boundary *y*_*k*_ = 0 or *y*_*k*_ = 0.5 we have

3.4*g* $$[\kappa_{pp}^{u}+(y_{p}-y_{k}){\hat{e}}_{p}]\cdot {\hat{e}}_{k}=0,\;y\in \Gamma_{ms}.$$

Alternatively, if the particle does not intersect the boundary *y*_*k*_ = 0 or *y*_*k*_ = 0.5, then we apply

3.4*h* $$[\kappa_{pp}^{u}+y_{p}{\hat{e}}_{p}-{\hat{\kappa }}_{pp}]\cdot {\hat{e}}_{k}=0,\;y\in \Gamma_{ms}$$

and

3.4*i* $$\int_{\Gamma_{\mathrm{ms},j}}{\hat{n}}_{0}^{\mathrm{ms}}\cdot \alpha_{pp}^{u}\cdot {\hat{e}}_{k}\;dy=0,$$

where ${\hat{\kappa }}_{pp}$ is an arbitrary constant vector that is unique to each particle. Similarly for the case *p*≠*q*, if the solid grains intersect with *y*_*k*_ = 0 or *y*_*k*_ = 0.5, we apply

3.4*j* $$\begin{array}{rl} & \left[\kappa_{pq}^{u}+\frac{y_{p}{\hat{e}}_{q}+y_{q}{\hat{e}}_{p}}{2}-{\hat{\kappa }}_{pq}\right]\cdot {\hat{e}}_{k}=0,\;y\in \Gamma_{\mathrm{ms},} \end{array}$$

3.4*k* $$\begin{array}{rl} & \int_{\Gamma_{\mathrm{ms},j}}{\hat{n}}^{\mathrm{ms}}\cdot \alpha_{pq}^{u}\cdot {\hat{e}}_{k}\;dy=0 \end{array}$$

3.4*l* $$\begin{array}{rl} \;\text{and}\; & \left[\kappa_{pq}^{u}+\frac{y_{p}{\hat{e}}_{q}+y_{q}{\hat{e}}_{p}}{2}\right]\cdot {\hat{e}}_{j}=0,\;j\neq k,\;y\in \partial \Gamma_{\mathrm{ms}}, \end{array}$$

where ${\hat{\kappa }}_{pq}$ is an arbitrary constant vector that is unique to each particle. It is possible that a single particle can intersect multiple coordinate boundaries and could have zero motion in the *y*_1_, *y*_2_ and *y*_3_ directions. Alternatively, it may have zero motion in one of these directions but not others. Hence, considerable care must be taken in defining the location of the grain boundaries in relation to the domain boundaries (figure 3).

The final cell problem describes the local deformation due to changes in pressure that move the soil away from the capillary equilibrium, i.e. *p*^a^_0_ − *p*^c^ − *p*^m^_0_≠0, where the capillary pressure *p*^*c*^ is defined in equation (3.1). We write this as

3.5*a* $$\begin{array}{rl} & \nabla_{y}\cdot \alpha^{p}=0,\;y\in \Omega_{m}, \end{array}$$

3.5*b* $$\begin{array}{rl} & \alpha^{p}=e_{y}(\kappa^{p})+\frac{\nu }{1-2\nu }(\nabla_{y}\cdot \kappa^{p})I,\;y\in \Omega_{m} \end{array}$$

3.5*c* $$\begin{array}{rl} \;\text{and}\; & {\hat{n}}^{\mathrm{am}}\cdot \alpha^{p}={\hat{n}}^{\mathrm{am}},\;y\in \Gamma_{\mathrm{am}}, \end{array}$$

where ***κ***^p^ is the displacement coefficient driven by a the absolute pressure. The symmetry boundary conditions are

3.5*d* $${\hat{e}}_{k}\cdot \kappa^{p}=0,\;y\in \partial y_{k}$$

and

3.5*e* $${\hat{e}}_{j}\cdot \alpha^{p}{\hat{e}}_{j}=0,\;j\neq k,\;y\in \partial y_{k}.$$

For a particle that intersects the boundary *y*_*k*_ = 0 or *y*_*k*_ = 0.5 we have

3.5*f* $$\kappa^{p}\cdot {\hat{e}}_{k}=0,\;y\in \Gamma_{\mathrm{ms}}.$$

Alternatively, if the particle does not intersect the boundary *y*_*k*_ = 0 or *y*_*k*_ = 0.5, then we apply the conditions

3.5*g* $$[\kappa^{p}-{\hat{\kappa }}^{p}]\cdot {\hat{e}}_{k}=0,\;y\in \Gamma_{\mathrm{ms}}$$

and

3.5*h* $$\int_{\Gamma_{\mathrm{ms},j}}{\hat{n}}^{\mathrm{ms}}\cdot \alpha^{p}\cdot {\hat{e}}_{k}\;dy=0,$$

where ${\hat{\kappa }}^{p}$ is a constant vector that is unique to each particle.

### (c) The averaged model

Using the results from the cell problems, the final stage of the homogenization procedure constitutes a volume average [31]. This approach was used to obtain a set of macroscopic equations for the solid displacement, the air pressure and the fluid pressure in the mixed phase, parametrized by the underlying geometry. In the original paper [31], this final stage of averaging resulted in 20 effective tensor quantities. Owing to the symmetry of the cell problems in this study, several of these parameters become identically zero. Hence, we present the final dimensionless parameters for the symmetric case. For the elastic displacement of the solid, the averaged force balance depends on

3.6*a* $$\begin{array}{rl} C_{ijkl}^{u} & =\frac{1}{∥\Omega_{m}∥}\int_{\Omega_{m}}\left[\frac{\partial \kappa_{kli}^{u}}{\partial y_{j}}+\frac{\partial \kappa_{klj}^{u}}{\partial y_{i}}+\delta_{ik}\delta_{jl}+\frac{\nu }{1-2\nu }(\delta_{ij}\delta_{kl}+\nabla_{y}\cdot \kappa_{kl}^{u}\delta_{ij})\right]dy\\ & \;-\frac{1}{∥\Omega_{m}∥}\int_{\Gamma_{\mathrm{ms}}}y_{i}{\hat{n}}_{0q}^{\mathrm{ms}}\left[\frac{\partial \kappa_{klq}^{u}}{\partial y_{j}}+\frac{\partial \kappa_{klj}^{u}}{\partial y_{q}}+\delta_{qk}\delta_{jl}+\frac{\nu }{1-2\nu }(\delta_{qj}\delta_{kl}+\nabla_{y}\cdot \kappa_{kl}^{u}\delta_{qj})\right]dy, \end{array}$$

3.6*b* $$\begin{array}{rl} C_{ij}^{p} & =\frac{1}{∥\Omega_{m}∥}\int_{\Omega_{m}}\left[\frac{\partial \kappa_{i}^{p}}{\partial y_{j}}+\frac{\partial \kappa_{j}^{p}}{\partial y_{i}}+\frac{\nu }{1-2\nu }\nabla_{y}\cdot \kappa^{p}\delta_{ij}\right]dy\\ & \;-\frac{1}{∥\Omega_{m}∥}\int_{\Gamma_{\mathrm{ms}}}y_{i}{\hat{n}}_{0q}^{\mathrm{ms}}\left[\frac{\partial \kappa_{q}^{p}}{\partial y_{j}}+\frac{\partial \kappa_{j}^{p}}{\partial y_{q}}+\frac{\nu }{1-2\nu }\nabla_{y}\cdot \kappa^{p}\delta_{qj}\right]dy \end{array}$$

3.6*c* $$\begin{array}{rl} \text{and}g^{\mathrm{eff}} & =g^{w}\left\{[\phi +\delta_{2}(1-\phi )]+\frac{∥\Omega_{a}∥}{∥\Omega_{m}∥}\delta_{3}+\frac{∥\Omega_{s}∥}{∥\Omega_{m}∥}\delta_{4}\right\}, \end{array}$$

where

3.7 $$∥\Omega_{r}∥=\int_{\Omega_{r}}\;dy\;y$$

for *r* = {a, m}. Here, *C*^u^_*ijkl*_ is the stiffness tensor and *C*^p^_*ij*_ is the pressure-induced stress coefficient, respectively. The parameters in the equation for the fluid pressure in the mixed phase are

3.8*a* $$\begin{array}{rl} & K_{ij}^{w}=\frac{1}{∥\Omega_{m}∥}\int_{\Omega_{m}}\delta_{ij}+\frac{\partial \omega_{j}^{m}}{\partial y_{i}}\;dy, \end{array}$$

3.8*b* $$\begin{array}{rl} & A_{ij}^{u}=\frac{1}{∥\Omega_{m}∥}\int_{\Omega_{m}}\nabla_{y}\cdot \kappa_{ij}^{u}\;dy \end{array}$$

3.8*c* $$\begin{array}{rl} \;\text{and}\; & A^{p}=\frac{1}{∥\Omega_{m}∥}\int_{\Omega_{m}}\nabla_{y}\cdot \kappa^{p}\;dy, \end{array}$$

where *K*^w^_*ij*_ is the effective water mobility, *A*^u^_*ij*_ and *A*^p^ are the increment of water content due to strain and pressure changes, respectively. Finally, the parameters in the equation for the air pressure are

3.9*a* $$\begin{array}{rl} K^{a} & =-\frac{1}{\delta_{1}∥\Omega_{a}∥}\int_{\Omega_{a}}\zeta_{k}⊗{\hat{e}}_{k}\;dy, \end{array}$$

3.9*b* $$\begin{array}{rl} B_{ij}^{u} & =\frac{1}{∥\Omega_{a}∥}\int_{\Gamma_{\mathrm{am}}}{\hat{n}}^{\mathrm{am}}\cdot \kappa_{ij}^{u}\;dy+\frac{1}{∥\Omega_{a}∥}\sum_{j}\int_{\Gamma_{\mathrm{as},j}}{\hat{n}}^{\mathrm{am}}\cdot \gamma_{ij}^{u}\;dy \end{array}$$

3.9*c* $$\begin{array}{rl} \;\text{and}\;B^{p} & =\frac{1}{∥\Omega_{a}∥}\int_{\Gamma_{\mathrm{am}}}{\hat{n}}^{\mathrm{am}}\cdot \kappa^{p}\;dy+\frac{1}{∥\Omega_{a}∥}\sum_{j}\int_{\Gamma_{\mathrm{as},j}}{\hat{n}}^{\mathrm{am}}\cdot \gamma^{p}\;dy. \end{array}$$

Here, *K*^a^_*ij*_ is the effective air mobility, *B*^u^_*ij*_ and *B*^p^ are the increment of air content due to strain and pressure changes, respectively. These equations provide a complete description of how the micro-scale geometry and mechanical properties influence the macro-scale mechanical properties of the poroelastic composite.

At this point, we consider the effect of solving equations (3.2) on a 1/8th geometry. We note that for the *k*-th cell problem, as ${\hat{e}}_{k}\cdot \zeta_{k}$ is even on all boundaries, we would need to multiply the result by a factor of 8 to account for the 7/8ths of the geometry not considered. However, as the effective parameters are all normalized by the cell volume, this factor of 8 will cancel out. In addition, as each of the remaining velocity components is odd about two boundaries, we find $\int_{\Omega_{a}}{\hat{e}}_{j}\cdot \zeta_{k}\;dy=0,\;j\neq k$. Hence, *K*^a^_*ij*_ is zero for *p*≠*q*. By a similar analysis as for (3.2) we find that *K*^w^_*ij*_, *C*^p^_*ij*_, *A*^u^_*ij*_ and *B*^u^_*ij*_ are zero if *i*≠*j*, and *C*^u^_*ijkl*_ is zero if *i*≠*k* and *j*≠*l*. In summary, the effective equations describe a three-phase poroelastic material and are parameterized by nine effective parameters. These are *g*^eff^ the effective gravitational force; *C*^u^_*ijkl*_, the macro-scale or effective stiffness tensor; *C*^p^_*ij*_ the stress coefficient for pressure variations about the capillary equilibrium point; *K*^w^_*ij*_ and *K*^a^_*ij*_, the water and air relative mobilities; and *A*^u^_*ij*_, *A*^p^, *B*^u^_*ij*_ and *B*^p^, the strain- and pressure-induced variations in water and air content.

## 4. lmage-based modelling

The symmetric cell problems (3.2)–(3.5) are in an appropriate form to be solved on an imaged geometry, as a result of the symmetry reduction we need consider only a subset of the upscaled parameters. They are *C*^u^_*ijkl*_ for *i* = *k* and *j* = *l*, *C*^p^_*ij*_ for *i* = *j*, *K*^w^_*ij*_ for *i* = *j*, *A*^u^_*pq*_ for *i* = *j*, *A*^p^, *K*^a^_*ij*_ for *i* = *j*, *B*^u^_*ij*_ for *i* = *j*, *B*^p^, ∥*Ω*_a_∥/∥*Ω*_m_∥ and ∥*Ω*_*s*_∥/∥*Ω*_m_∥.

### (a) Numerical implementation

**Figure 4.**
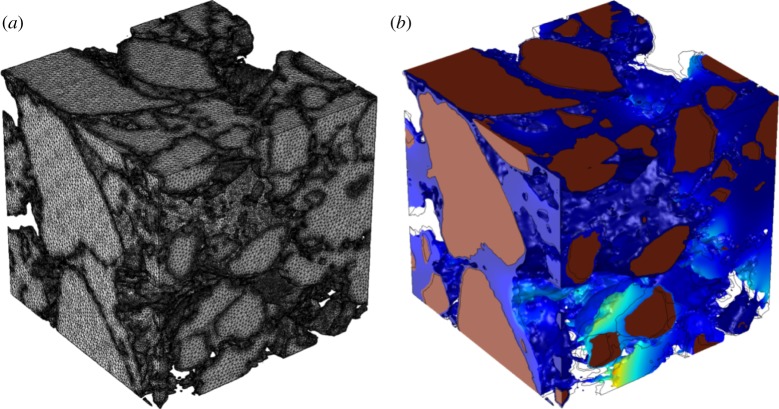
Typical domain used for simulation. (*a*) Computational mesh generated from X-CT images. (*b*) Typical solution showing magnitude of pressure-induced displacement. For illustrative purpose, the mesh has been warped by an amount proportional to the displacement, the outline of the original domain has been added as a guide to the eye. (Online version in colour.)

We solve equations (3.2)–(3.5) for each of the three soil treatments with six replicates. As a first step, we generate a computational mesh based on the X-ray CT images. Mesh generation is achieved using Simpleware 2016.09, a commercial software package designed to generate computational and surface meshes. The mesh is generated directly from the segmented images and allows generation of a volume mesh for the mixed phase and unique identifiable boundaries for the solid particles. The mesh was generated using the FE-FREE algorithm to allow Simpleware the maximum control over the mesh elements while minimizing memory requirements. A typical mesh and solution to equation (3.5) is shown in figure 4.

To ensure that the computational domain comprised a sufficiently large volume to be representative of the soil structure, we considered a sequence of meshes of different volumes. Specifically we considered meshes of side lengths of 100–400 voxels increasing in steps of 20 voxels. This corresponds to a smallest mesh of total side length 0.16 mm and a largest mesh of 0.64 mm. Owing to the symmetry reduction, this means we are modelling an effective soil volume of maximum side length 1.28 mm, which is larger than the largest soil particles permitted through the sieve during soil preparation.

We implement the cell problems in equations (3.3)–(3.5) using Comsol Multiphysics 5.2a, a commercial finite-element solver. Owing to the complexity of the mesh required for the solution of Stokes equations, the cell problem in equation (3.2) is solved using OpenFOAM, an open source volume of fluid solver. While, in principle, all the cell problems could be solved in OpenFOAM, existing solvers are not set up for this and would require further work to develop and validate. Hence, we opted to solve the majority of cell problems in Comsol and only used OpenFOAM for cell problem (3.2). The implementation of these equations on the imaged geometries is non-trivial due to the complexity of the underlying physics. Each of the solid particles is able to move with a unique displacement meaning that it must be associated with a different physical condition. From a meshing perspective, this means that each individual solid particle had to be attributed a unique label. Owing to limitations in the software used, the maximum number of labels which could be defined is 200. This was not a sufficiently large number to ensure that the solutions to the cell problems had converged to a representative value. Hence, for the smaller cell problems (side length less than 300 voxels), we assigned each of the particles a unique boundary condition. For all cell problems (side length 100–400 voxels), we assumed that the solid phases could be represented as a compressible elastic material with a stiffness which was much greater than the mixed phase. We compared the output from these two methods for meshes with side length 100–300 voxels and found the differences to be minimal (less than 1% in all cases tested).

To ensure that each particle has the correct physics applied to it in a consistent way the model is developed using the Comsol-Matlab Live-Link, a toolkit which enables the finite-element functionality of Comsol to be accessed and scripted via Matlab. Once the meshes had been generated and an appropriate set of physics had been prescribed, the models were solved on the Iridis 4 super computing cluster and, for meshes with side length greater than 300 voxels, on two bespoke high memory machines each with greater than 500 Gb of RAM.

### (b) Convergence

We consider the convergence of all the homogenized parameters in the averaged equations (3.6), (3.8) and (3.9). This is essential as, due to the presence of three phases, some of the parameters will converge much more rapidly than others. In addition, as the soils are highly heterogeneous, it is possible that the behaviour of some replicates may not converge within the size range considered. Hence, we develop a set of criteria which allows us to eliminate replicates which have not converged.

We consider that a generic averaged parameter *u* for a specific replicate has converged with respect to the domain size *L* if two criteria are met. Firstly, we do not see large variation in the value of *u* relative to the average value. In other words, we expect to be able to fit a smooth function *f*(*L*) to the data *u*(*L*) which describes the convergence to a reasonable degree of accuracy. We choose to fit the function *f*(*L*) = *a* + *b*e^−*cL*^, where *a*, *b* and *c* are fitting parameters. In the limit *L* → ∞, we observe that *f*(*L*) → *a*. Hence, our criterion is that, for a set of discrete points *L*_*j*_ the root mean squared difference between *f*(*L*_*j*_) and *u*(*L*_*j*_) is sufficiently small with respect to the steady value *a*, i.e.

4.1 $$\sqrt{\frac{\sum_{j=0}^{N}{\left[f(L_{j})-u(L_{j})\right]}^{2}}{f{\left(L_{N}\right)}^{2}}}<f_{\mathrm{tol}},$$

where we choose *f*_tol_ = 0.05 allowing a maximum root mean squared difference of 5% between *f*(*L*) and *u*(*L*). This criterion is not an absolute criterion on convergence, rather it is a minimum condition which the replicates have to meet to be considered as behaving sufficiently well that they may converge within the range of sample sizes considered.

To ensure that the replicates are representative, we also require that the values converge sufficiently quickly with respect to the sample size *L*. We can express this through the requirement that the decaying part of *f*(*L*) is small with respect to the final value of *f*(*L*), hence we require

4.2 $$\frac{|be^{-cL_{N}}|}{|a|}<\delta f_{\mathrm{tol}},$$

where we choose *δf*_tol_ = 0.1, i.e. we require that the parameter has converged to within 10% of its final value within the size range considered. If a replicate meets these two criteria, then we consider it to have converged to a representative value and include it in the analysis. If however, either of the criteria are not met, then we assume that the replicate has not converged and do not include it in the analysis.

We calculate convergence of each replicate for all the parameters considered as some properties may converge more readily than others. For each parameter, we plot the average of the converged replicates and give the standard deviation as error bars, taking into account the converged replicates only. It is likely that, due to differences in preparation, the mixed phase will have different poro-elastic properties between the different treatments. These differences will manifest themselves in the parameters *G*, *k* and *ν*, the shear modulus, permeability and drained Poisson ratio of the mixed phase, respectively. These three parameters are dependent on the properties of the underlying elastic solid and fluid that comprise the mixed phase, and the underlying geometrical arrangement of these phases [18,19]. Of these parameters the only one which appears in the cell problems is the drained Poisson ratio. However, it is known that the majority of clay soils exhibit a high drained Poisson ratio in the range 0.4–0.5 [44], hence, we have assumed that this will be the same in the three different treatments and chosen a value of *ν* = 0.45.

The converged results for all effective parameters were assessed by analysis of variance. The probability of significance *P*, with a threshold value of (*P* < 0.05) was calculated and is used as a measure of significance of the results obtained.

## 5. Results and discussion

We start by considering the convergence of each parameter. We have plotted the convergence of all parameters either as part of the paper, or as part of electronic supplementary material, S2. The number of converged replicates for each parameter is noted in the figure captions, unless otherwise stated the maximum number of converged replicates is six in each case. By considering the volume fractions in each treatment, we observe evidence of convergence (based on the criteria (4.1) and (4.2)) before the largest *L* considered, i.e. *L* = 0.64 mm, electronic supplementary material, figure S2.1. While this may seem small we note that, due to the symmetry boundary conditions considered, a simulated soil volume of 0.64 mm corresponds to a soil sample size of 1.28 mm, which is larger than the maximum particle size present in these samples. The wet treatments have a significantly higher mixed phase volume fraction than the drier treatment. The convergence rate is seen to be much slower for the wet compacted sample than the other two which converged for a lower *L*.

**Figure 5.**
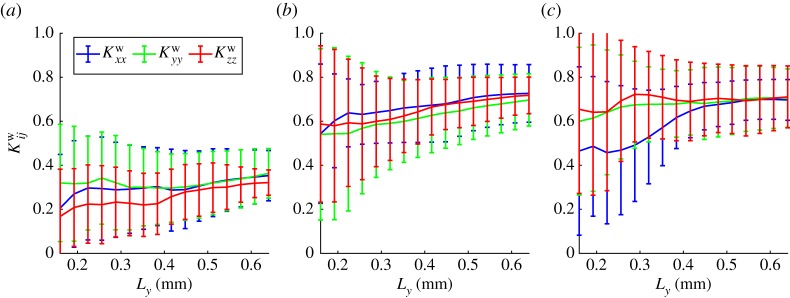
The effective water mobility, *K*^w^_*ij*_ for *i* = *j*. Showing (*a*) the dry compacted phase (minimum five replicates), (*b*) the wet compacted phase (minimum four replicates) and (*c*) the wet uncompacted phase (minimum four replicates, maximum five replicates). The error bars show the standard deviation across the converged replicates. (Online version in colour.)

**Figure 6.**
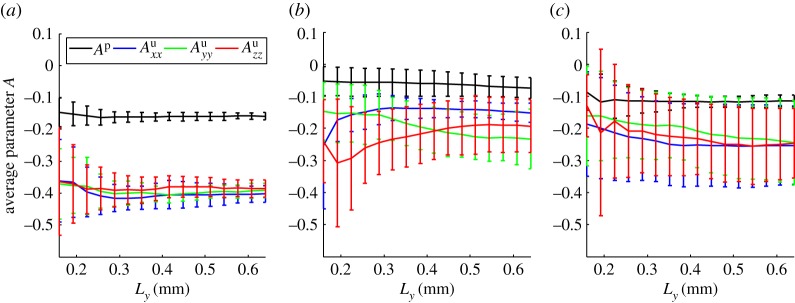
The effective parameters for the change in water volume due to pressure variations (*A*^p^) and strain variations (*A*^u^_*ij*_, *i* = *j*). Showing (*a*) the dry compacted phase (minimum five replicates), (*b*) the wet compacted phase (minimum four replicates) and (*c*) the wet uncompacted phase (minimum five replicates). The error bars show the standard deviation across the converged replicates. (Online version in colour.)

The convergence of the parameters (3.8) is shown in figures 5 and 6. The effective water mobility (figure 5), is seen to converge for each of the three treatments and is significantly lower in the drier compact sample than the two wet samples and corresponds to the volume fraction of the mixed phase, electronic supplementary material, figure S2.1. We recall that the dimensional permeability will be given by $\tilde{K}=kK_{ij}^{w}$ and will depend on the permeability of the uncompressed mixed phase. This parameter is difficult to measure directly and is likely to be different between the three treatments. In fact, the results presented here could be seen as a means to infer the permeability of the mixed phase when combined with a macro-scale measurement of the soil permeability. The parameters *A*^p^ and *A*^u^_*ij*_ describe the variation in fluid content due to variation in pressure and strain, respectively. These parameters converge well for the geometry sizes considered here. The effect of strain is seen to dominate over pressure. However, this is only significant in the dry compact case where a unit change in strain will produce an effect two and a half times larger than a unit change in pressure.

**Figure 7.**
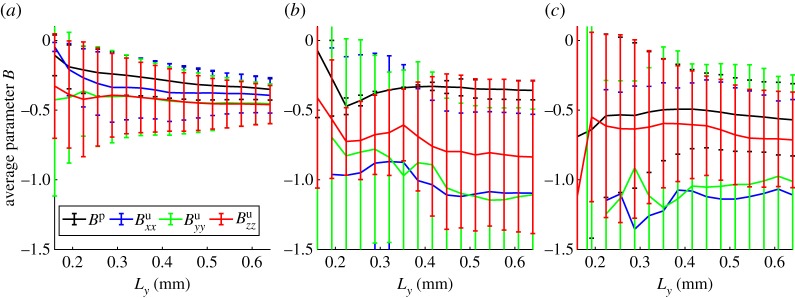
Effective parameter describing the change in air-filled pore volume as a result of changes in strain (*B*^u^_*ij*_ for *i* = *j*) and changes in pressure (*B*^p^). Showing (*a*) the dry compacted phase (minimum four replicates), (*b*) the wet compacted phase (minimum three replicates, maximum five replicates) and (*c*) the wet uncompacted phase (minimum four replicates). The error bars show the standard deviation across the converged replicates. (Online version in colour.)

The parameters used in equation (3.9) are given in electronic supplementary material, figures S2.2 and 7. Electronic supplementary material, figure S2.2 shows the air mobility for the three different treatments which is calculated using the parameter *δ*_1_ (equation (3.1)) in which we used the values *k* = 10^−10^ m^2^ [45], *μ*^w^ = 8.9 × 10^−4^ Pa s and *μ*^a^ = 1.8 × 10^−5^ Pa s. The convergence of this parameter was much poorer than that of the other parameters, hence, these results are not discussed in detail. The parameters *B*^p^ and *B*^u^_*ij*_, which describe the change in air volume due to pressure variation and strain variation, respectively, are shown in figure 7. The convergence in this case is much better than for the air mobility. The effect of changes in pressure and the effect of changes in strain do not show significant differences for all cases. The only significant difference is between the dry and wet compacted cases.

**Figure 8.**
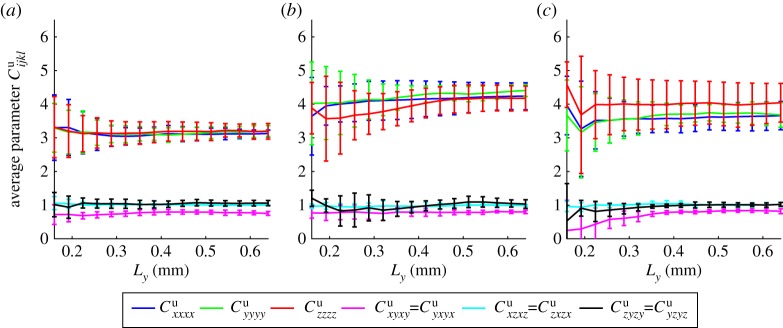
Effective parameter describing the change in stress due to a change in strain (*C*^u^_*ijkl*_ for *i* = *k*, *j* = *l*). Showing (*a*) the dry compacted phase (minimum six replicates), (*b*) the wet compacted phase (minimum six replicates) and (c) the wet uncompacted phase (minimum five replicates). The error bars show the standard deviation across the converged replicates. (Online version in colour.)

Finally, we consider the effective parameters *C*^p^_*ij*_ and *C*^u^_*ijkl*_ which describe how pressure and strain variations affect the stress tensor of the soil. We see strong convergence for all of the different soil treatments in this case and, due to the large drained Poisson ratio, we see that the diagonal components of *C*^u^_*ijkl*_ (*i* = *j* = *k* = *l*) significantly dominate the behaviour of this equation. Hence, we present only the stiffness tensor *C*^u^_*ijkl*_ (figure 8). The pressure-induced stress coefficient is plotted in electronic supplementary material, S2. The effect of pressure changes, compared to changes in dilation, is seen to be small resulting in a material whose effective elastic properties are those of a relatively incompressible elastic solid. This is particularly evident in the case of the wet compacted soil samples, suggesting that the majority of the compressibility comes from reduction in air volume. Overall the only significant difference observed between treatments was for the dry and wet compacted samples. For the majority of measurements, compaction did not significantly alter the soil properties.

We observe that some of the effective parameters, *C*^u^_*ijkl*_ and *C*^p^_*ij*_ for example, have clearly converged within the range of sample volumes considered. However, if we look at the *K*^a^_*ij*_, *B*^u^_*ij*_ or *B*^p^ we see that there is little evidence of the samples converging to a representative value. In addition, we note that there is substantial heterogeneity between the different samples, which is indicated by the size of the error bars. In other words, the parameters have converged, but the different replicates have each converged to different values. It is possible that this heterogeneity would be removed if the representative volumes of soil used were larger. This was not practical in this case as, due to the need for high resolution, the soil samples used were relatively small. While it would be possible to prepare larger samples, obtaining X-CT scans of larger samples requires a reduction in resolution which would make it harder to characterize the different phases. In principle multiple scans could be taken, some larger scans at a coarser resolution to allow the air phases to be characterized and some smaller region of interest scans at a finer resolution to enable the fine detail to be captured. However, the sizes required for this would depend on the soil types used and the preparation methods [34].

Often in homogenization studies, the link between the micro- and macro-scale properties is dependent only on the geometry [20,21]. Here however, there are several small-scale parameters which are unknown; they are the shear modulus *G*, the drained Poisson ratio *ν* and the permeability *k* of the mixed phase. The dimensionless parameters presented in this paper are independent of *G*. We note that this does not mean that *G* has no effect on the physical properties of the soils, simply that it scales out of the cell problems presented in §3b. The dimensionless air mobility depends on *δ*_1_ (equation (3.1)) and is therefore dependent on *k*; the remaining dimensionless parameters are not affected by the value of *k*. The drained Poisson ratio has an effect on the parameters *C*^u^_*ijkl*_, *C*^p^_*ij*_, *B*^p^ and *A*^p^. Hence, this method cannot predict absolute values of different soils without a direct measure of the micro-scale physical parameters. However, it can determine the link between the properties observed on the micro-scale and the properties observed on the macro-scale. For example, when combined with macro-scale measurements it could be used to reverse engineer the properties of the soil constituents by solving an inverse problem. This could, in turn, lead to the potential to design and/or optimize soil and other porous structures. For the same reasons, while experimental validation is an important step it is not necessarily practical to do this in this study.

## 6. Conclusion

In this paper, we have applied the theory derived in [31] to calculate the link between the micro-scale and macro-scale poroelastic properties of a three-phase poroelastic medium. We considered three different soil treatments combining two different moisture treatments and two different initial levels of compaction. We found that the effective properties of the soils were unaffected by the compaction. However, changing the moisture content significantly altered the hydraulic and mechanical properties of the soils.

The soils were assumed to be at equilibrium when imaged. In this work, physical parameters such as distribution of mineral sizes and contact angles were determined implicitly through imaging. In other words, the contact angles and particle size distributions were never measured explicitly. Rather their effect was included in the calculations through the imaged geometry. The analysis presented here amounts to finding the macro-scale poroelastic properties of the soils by calculating how they respond to different perturbations. Hence, the results are accurate only at the saturation at which the soils were scanned. In principle, multiple scans could be taken to calculate the properties at a range of saturation values. By combining this theory with an explicit model for air and water interaction, such as the one used in [24], this theory could be extended across the whole saturation range without the need for multiple X-CT scans. This represents a significant challenge, which would also allow the dependence of features such as the air–water contact angle to be incorporated explicitly into the analysis.

We have shown that this method can be used to determine differences in the macro-scale poroelastic properties of a soil if the micro-scale properties are the same. In particular, we have assumed that the drained Poisson ratio and permeability of the mixed phase was the same in all cases. In addition, we have implicitly assumed that the shear modulus of the mixed phase remains constant. However, this parameter does not come into the dimensionless equations solved in this paper. Hence, in principle it could be considered different between samples. Owing to the dependence on the micro-scale parameters, the method described cannot determine macro-scale soil properties on its own. Rather, it is seen as a method to probe and understand link between the observed properties on the micro- and macro-scales. The multiscale nature of this technique means that it is ideally suited to rhizosphere scale imaging experiments and simulations [34,46].

For the samples considered in this paper, we found that the main factor affecting the macroscopic properties of the soils is the mixed phase volume fraction. We found that the drier samples, in which the mixed phase occupied a significantly smaller volume, had a lower effective water mobility and a higher air mobility. In addition, variations in strain had a much larger effect on the overall change in water and air volume for the drier samples (figures 6 and 7), and the effective stress tensor was more sensitive to the absolute pressure than the corresponding wetter samples (electronic supplementary material, figure S2.3). In comparison, we found that the differences between a compacted and an uncompacted soil were not significant suggesting that the air content and connectivity, i.e. the ability to squeeze air out of the soil, is a major contributing factor in the compression of soils in addition to the compaction of the mixed phase itself. We note however, that the range of compaction used in this paper is small and we would expect that a large initial compaction could result in a much greater variation in soil poroelastic properties.

Looking forward, the models developed and applied in this paper have been validated numerically and mathematically. However, experimental validation is required before the models can be considered truly predictive. This is a non-trivial step as the mixed phase properties will need to be measured independently of the macroscopic properties. The results presented in this paper will serve to guide such a study and provides numerical results, which could be used as a first step in such a validation.

Overall, this technique provides the means to probe the effects of micro-scale parameters and geometry on macro-scale properties. In addition to probing the properties of different soil structures, this technique will enable the possibility of designing soils composed of different soil fractions with the aim of obtaining a specific set of macro-scale structural parameters. Specifically, results obtained from this study could be used in combination with micro-scale measurements of plant soil interaction to create designer environments for plant growth [47], and to guide agricultural practice relating to soil compaction [1].

## Supplementary Material

Supplementary 01

## Supplementary Material

Supplementary 02

## Data accessibility

A full set of raw data, raw meshes and an example set of models is included in the electronic supplementary material. With all data supporting this study are openly available from the University of Southampton repository at https://doi.org/10.5258/SOTON/D0445 [48].
