# Supplementary material for: Determination of macro-scale soil properties from pore scale structures: image-based modelling of poroelastic structures
Source: Proc Math Phys Eng Sci. 2018 Jul 11;474(2215):20170745. doi: 10.1098/rspa.2017.0745 (PMC6083241; doi:10.1098/rspa.2017.0745)
Supplement: Supplementary 01 [file rspa20170745supp1.pdf]

S1. The mathematical model

In this supplementary material we provide more details of the mathematical model derivation and symmetry reduction used in the main paper.

(a) The micro-scale equations

The first stage of the homogenisaiton procedure was to consider a microscopic description of the soil physical properties. We considered the soil domain to be composed of a solid phase $\mathfrak{B}_s$, an air phase $\mathfrak{B}_a$ and a mixed phase $\mathfrak{B}_m$. The phase boundaries are defined as $\partial\mathfrak{B}_{as,j}$, $\partial\mathfrak{B}_{ms,j}$ and $\partial\mathfrak{B}_{am}$ for the air–solid, mixed–solid and air–mixed boundaries respectively. The mixed phase is composed of water and smaller mineral phases and is mathematically described as a poroelastic mixture [1–4]. We make no assumptions about the volume ratio of water to smaller mineral phases within the mixed phase. In reality, the mixed phase of a drier soil would have a much higher fraction of solid than water, whilst a wetter soil would have a much larger fraction of liquid than solid. We also define $\hat{\boldsymbol{n}}^{am}$ as the unit normal vector on the air–mixed phase interface pointing into the mixed phase domain, $\hat{\boldsymbol{n}}^{as}$ as the unit normal vector on the air–solid interface pointing into the solid domain, and $\hat{\boldsymbol{n}}^{ms}$ as the unit normal vector on the mixed phase–solid interface pointing into the solid domain.

The three phase poroelastic material is described in terms of solid displacements, and fluid pressures and velocities. We denote the solid displacements $\tilde{\boldsymbol{u}}^r$ where $r = \{a, m, s\}$ denotes the air, mixed and solid phase respectively. The fluid pressures are denoted $\tilde{p}^a$ and $\tilde{p}^m$ for the air and mixed phases respectively. The fluid and solid velocities can be calculated through the material derivative $\tilde{\boldsymbol{v}}^r = \partial_{\tilde{t}}\tilde{\boldsymbol{u}}^r + \tilde{\boldsymbol{v}} \cdot \tilde{\boldsymbol{\nabla}}\tilde{\boldsymbol{u}}^r$ [4].

The first step in deriving the averaged behaviour of the soils was to non-dimensionalise the model using $\tilde{\boldsymbol{u}}^r = [\boldsymbol{u}]\boldsymbol{u}^r$, $\tilde{t} = [t]t$, $\tilde{\boldsymbol{v}}^r = ([\boldsymbol{u}]/[t])\boldsymbol{v}^r$, $\tilde{\boldsymbol{x}} = [\boldsymbol{x}]\boldsymbol{x}$ and $\tilde{p}^r = [p]p^r$. We are interested in considering a system of typical size L_x with periodic micro-structure $L_y \ll L_x$, such that the scaling ratio $\epsilon = \frac{L_y}{L_x} \ll 1$. We want to obtain a model which is valid on the macroscopic length scale L_x and has displacement of typical size L_y. The final equations we derive are valid up to $O(\epsilon^0)$, *i.e.*, they will have error of size ϵ associated with them. Hence, we non-dimensionalise with respect to these length scales,

$$[\boldsymbol{x}] = L_x, \quad [\boldsymbol{u}] = L_y, \quad [p] = \frac{G[\boldsymbol{u}]}{L_x}, \quad [t] = \frac{\mu^w L_x^2}{kG}, \tag{S1.1}$$

where G is the shear modulus of the mixed phase, μ^w is the viscosity of water, and k is the permeability of the mixed phase. We note that in the general case derived in [5], the interaction between the mixed phase and the adjacent phases was described by a generalised Navier slip condition, parameterised by a pair of slip lengths $\tilde{\lambda}^s$ and $\tilde{\lambda}^a$ on the solid and air boundaries respectively. After homogenisation it was found that the parameter $\tilde{\lambda}^a$ does not play a role in determining the macroscopic properties of the soil [5]. Hence, we do not consider it in this paper. The parameter $\tilde{\lambda}^s$ is the slip length of the mixed phase acting on the solid particles. Physically, this is a function of the porosity, particle size, particle density, and the viscosity of the fluid. For a low permeability mixed phase, there will only be a thin water film between the solid component of the mixed phase and the larger solid grains. Hence, we would expect $\tilde{\lambda}^s \ll 1$. For a higher permeability mixed phase, there would be a larger water layer resulting in a larger $\tilde{\lambda}^s$. This situation is somewhat analogous to the movement of water through a pipe with porous walls; in which case the appropriate boundary condition would be the Beavers and Joseph condition [6], where the slip length associated with a permeable medium is $\tilde{\lambda}^s = \alpha\sqrt{k}$ and α is a constant of order 1. Assuming a similar relationship holds here, the slip length will only become important if $\tilde{\lambda}^s/L_x \gtrsim 1$. Typically, we are interested in considering soils on the length scale $L_x \sim 10\,\mathrm{m}$ and mixed phases with permeability in the range $k \lesssim 10^{-10}\,\mathrm{m}^2$. Hence, we chose to set $\tilde{\lambda}^s$ to zero and not consider it further in this analysis, further details on the validity range for these equations can be found in [5]. This scaling results in the following set of non-dimensional equations for the

rspa.royalsocietypublishing.org Proc R Soc A 0000000

mixed phase

$$\nabla \cdot \sigma^m - \nabla p^m = g^w \left[\phi + \delta_2(1-\phi)\right] \hat{e}_3, \qquad x \in \mathfrak{B}_m, \qquad \text{(S1.2}a\text{)}$$

$$\nabla \cdot v^m = \nabla \cdot \left(\nabla p^m + g^w \hat{e}_3\right), \qquad x \in \mathfrak{B}_m, \qquad \text{(S1.2}b\text{)}$$

where the effective stress is given by

$$\sigma^m = e(u^m) + \frac{\nu}{1-2\nu}\left(\nabla \cdot u^m\right) I, \qquad x \in \mathfrak{B}_m. \qquad \text{(S1.2}c\text{)}$$

Hence, the total stress in the mixed phase is given by $\sigma = \sigma^m - I p^m$. The solid phase is described through rigid body translations,

$$\sum_{i=1}^{2} \left(\hat{\tau}_i \cdot e(u^s) \cdot \hat{e}_j\right)^2 = 0, \qquad x \in \mathfrak{B}_{s,j}, \qquad \text{(S1.2}d\text{)}$$

$$\int_{\partial \mathfrak{B}_{ms,j}} \hat{n}^{ms} \cdot \sigma^{s,j}\, dx + \int_{\partial \mathfrak{B}_{as,j}} \hat{n}^{as} \cdot \sigma^{s,j}\, dx = -\epsilon g^w \delta_4 \int_{\mathfrak{B}_{s,j}} 1\, dx \hat{e}_3, \qquad \text{(S1.2}e\text{)}$$

and the air phase is described as a Stokes incompressible fluid,

$$\nabla \cdot \sigma^a - \nabla p^a = g^w \delta_3 \hat{e}_3, \qquad x \in \mathfrak{B}_a, \qquad \text{(S1.2}f\text{)}$$

$$\nabla \cdot v^a = 0, \qquad x \in \mathfrak{B}_a, \qquad \text{(S1.2}g\text{)}$$

$$\sigma^a = \epsilon^2 \delta_1 e(v^a), \qquad x \in \mathfrak{B}_a, \qquad \text{(S1.2}h\text{)}$$

$$v^r = \frac{\partial u^r}{\partial t} + \epsilon v^r \cdot \nabla u^r, \qquad \text{(S1.2}i\text{)}$$

for $r = \{m, s, a\}$. Here $e(u^m) = (\nabla u^m) + (\nabla u^m)^T$ is the strain and I is the identity matrix, ν is the drained Poisson ratio, ϕ is the porosity of the mixed phase, $\hat{\tau}_i$ are vectors tangent to the solid particle surface, and $\sigma^{s,j}$ is the stress tensor on the j–th solid particle. We write the boundary conditions on the mixed-solid interface as

$$\hat{n}^{ms} \cdot \left(\nabla p^m + g^w \hat{e}_3\right) = 0, \qquad x \in \partial \mathfrak{B}_{ms}, \qquad \text{(S1.2}j\text{)}$$

$$\hat{n}^{ms} \cdot \sigma^s = \hat{n}^{ms} \cdot \sigma^m - \hat{n}^{ms} p^m, \qquad x \in \partial \mathfrak{B}_{ms}, \qquad \text{(S1.2}k\text{)}$$

$$u^m - u^s = 0, \qquad x \in \partial \mathfrak{B}_{ms}. \qquad \text{(S1.2}l\text{)}$$

On the air–mixed phase boundary we write equations as

$$\hat{n}^{am} \cdot \left(\nabla p^m + g^w \hat{e}_3\right) = 0, \qquad x \in \partial \mathfrak{B}_{am}, \qquad \text{(S1.2}m\text{)}$$

$$\hat{n}^{am} \cdot \sigma^a - \hat{n}^{am}(p^a - p^c) = \hat{n}^{am} \cdot \sigma^m - \hat{n}^{am} p^m, \qquad x \in \partial \mathfrak{B}_{am}, \qquad \text{(S1.2}n\text{)}$$

$$v^m - v^a = 0, \qquad x \in \partial \mathfrak{B}_{am}. \qquad \text{(S1.2}o\text{)}$$

Finally on the air–solid interface we write the boundary conditions

$$v^a = v^s, \qquad x \in \partial \mathfrak{B}_{as}, \qquad \text{(S1.2}p\text{)}$$

$$\hat{n}^{as} \cdot \sigma^a - \hat{n}^{as} p^a = \hat{n}^{as} \cdot \sigma^s, \qquad x \in \partial \mathfrak{B}_{as}. \qquad \text{(S1.2}q\text{)}$$

Here the non-dimensional constants are

$$\delta_1 = \frac{\mu^a k}{\mu^w L_y^2}, \quad \delta_2 = \frac{\rho^m}{\rho^w}, \quad \delta_3 = \frac{\rho^a}{\rho^w}, \quad \delta_4 = \frac{\rho^s}{\rho^w}, \quad g^w = \frac{\rho^w g L_x^2}{G L_y}, \quad p^c = \frac{\gamma \kappa L_x}{G L_y}, \tag{S1.3}$$

where ρ^w is the water density, ρ^m is the density of the solid part of the mixed phase, g is the acceleration due to gravity, κ is the mean curvature of the air–water interface, and γ is the surface tension. In order to confirm our assumption, that the soils are approximatly capillary static, we can estimate the capillary number (on the micro-scale) as $Ca = (kG)/(L_y\gamma) \approx 1.38 \times 10^3$, assuming $k = 10^{-10}\,\mathrm{m}^2$, $G = 10^8\,\mathrm{Pa}$, $L_y = 10^{-3}\,\mathrm{m}$, and $\gamma = 0.72 \times 10^{-3}\,\mathrm{Pa\,m}$. Hence, we note that capillary forces dominate over viscous forces and our assumption that the soil is capillary static is reasonable. We note this estimate of Ca is likely to be an overestimate as the length scale used is that of the representative volume rather than the actual pore size (which will be smaller).

(b) Cell problems

In order to derive an averaged model for the poro-elastic behaviour of soils the method of homogenisation was applied [5]. The approach involved deriving a set of cell problems which capture the effect of the underlying geometry on the mechanical and fluid properties of the soil. Here our approach differs slightly from the one described in [5]. The key assumption used in [5] was that the geometry is periodic, *i.e.*, it is composed of regularly repeating units. In reality soils will not have a perfectly periodic structure. Hence, we follow the approach of enforcing periodicity via reflection of the imaged geometry [7].

The aim, for each cell problem, is to obtain a reflected cell problem based on the reflections $y_k \to -y_k$ for $k = \{1, 2, 3\}$. The reflected cell problems are identical to the original cell problems under an appropriate translation of the dependent variable, ($u \to -u$ for example). By finding these translations we can infer that if the system is invariant under the translation $y_k \to -y_k$ and $u \to -u$, then u must be odd about the plane of reflection. Hence, an appropriate boundary condition would be $u = 0$ on this plane. By repeating this approach for all directions we can infer a set of boundary conditions to impose on the edges of our domain which respect the symmetry of the underlying cell problems.

This symmetry reduction allows us to overcome the lack of periodicity in the imaged soils [7,8]. However, it comes at the price of forcing us to make assumptions regarding the structure and anisotropy of the imaged geometry. Clearly the soil image, Figure 1, is not composed of regularly repeating units. Hence, enforcing periodicity changes the soil structure. However, assuming that the soil structure is sufficiently homogeneous, enforcing periodicity by reflection will only create a significantly altered soil structure on the boundaries of the geometry. As the volume considered is increased the total volume fraction that is altered by the reflection is reduced. Hence, by increasing the volume of soil considered we would expect the effective properties measured to converge to those of the soil sample.

From a physical perspective, a periodic geometry composed of arbitrary repeating units could be isotropic, anisotropic or even chiral. However, if we assume that the representative unit has reflective symmetry in the three principal Cartesian directions then we cannot construct a chiral material. In addition the only anisotropic materials which respect these symmetries are those which are already aligned along the principal coordinate axes about which we make our reflections. In other words, we are only able to detect the diagonal components of any anisotropic material. These assumptions are necessary in order to obtain an appropriate representation of the soil on which the cell problems can be solved. In addition we have no reason to believe that these soil samples will have a chiral structure or that they will exhibit any off-axes anisotropy. It is possible that, as gravity acts on the soil in a single direction (aligned with the coordinate axes), there will be some variation between the effective parameters in the y_1, y_2 and y_3 directions. The symmetry reduction we have considered will allow us to detect this difference.

The cell problems derived in [5] describe the Darcy flow of air through the porespace, the impeded Darcy flow of water in the mixed phase, the local stiffness, and the pressure induced

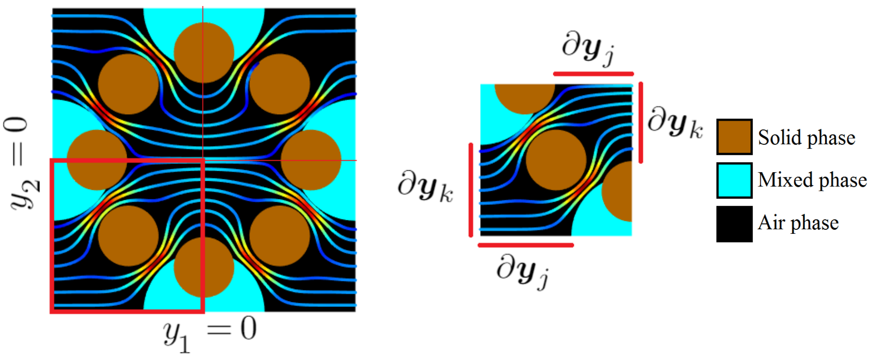

Figure S1.1. Symmetry reduction for air flow in three phase poroelastic soil. The left hand image shows the full geometry with periodic boundary conditions, the right hand image shows the corresponding reduced image with appropriate symmetry conditions applied on the boundaries ∂y_k and ∂y_j. The streamlines are coloured with the velocity magnitude with red corresponding to faster flow and blue corresponding to slower flow.

stress. In order to illustrate the symmetry reduction we start with the Darcy flow of air through the porespace, illustrated schematically in Figure S1.1. The cell problem derived in [5] comes from the first order expansion of Stokes' equations

$$\delta_1 \nabla_y^2 \boldsymbol{v}_0^a - \boldsymbol{\nabla}_y p_1^a = \boldsymbol{\nabla}_x p_0^a, \qquad \boldsymbol{y} \in \Omega_a^{1/8}, \tag{S1.4a}$$

$$\boldsymbol{\nabla}_y \cdot \boldsymbol{v}_0^a = 0, \qquad \boldsymbol{y} \in \Omega_a^{1/8}, \tag{S1.4b}$$

$$\boldsymbol{v}_0^a = 0, \qquad \boldsymbol{y} \in \Gamma_{am}^{1/8} \cup \Gamma_{as}^{1/8}, \tag{S1.4c}$$

(S1.4d)

combined with the observation that $p_0^a \sim p_0^a(\boldsymbol{x})$, *i.e.*, p_0^a is independent of the microscale variable, see [5] for full details. Hence, by writing

$$\boldsymbol{v}_0^a = \frac{1}{\delta_1} \sum_{k=0}^{3} \boldsymbol{\zeta}_k \partial_{x_k} p_0^a, \quad p_1^a = \sum_{k=0}^{3} \omega_k^a \partial_{x_k} p_0^a, \tag{S1.5}$$

where $\boldsymbol{\zeta}_k$ and ω_k^a are the local velocity and pressure coefficients driven by a large scale pressure gradient in the air phase. These can be determined by solving the cell problem

$$\nabla_y^2 \boldsymbol{\zeta}_k - \boldsymbol{\nabla}_y \omega_k^a = \hat{\boldsymbol{e}}_k, \qquad \boldsymbol{y} \in \Omega_a^{1/8}, \tag{S1.6a}$$

$$\boldsymbol{\nabla}_y \cdot \boldsymbol{\zeta}_k = 0, \qquad \boldsymbol{y} \in \Omega_a^{1/8}, \tag{S1.6b}$$

$$\boldsymbol{\zeta}_k = 0, \qquad \boldsymbol{y} \in \Gamma_{am}^{1/8} \cup \Gamma_{as}^{1/8}, \tag{S1.6c}$$

where periodic boundary conditions were assumed. For the imaged geometries we assume that the imaged geometry comprises $1/8$ th of the representative volume of soil. The total geometry can be reconstructed by reflecting the imaged data in $y_1 = 0$, $y_2 = 0$ and $y_3 = 0$. By imposing the translations $y_a \to -y_a$, for $a = \{1, 2, 3\}$, we derive the following symmetry boundary conditions

$$\omega_k^a = 0, \ \frac{\partial}{\partial y_k} (\hat{\boldsymbol{e}}_k \cdot \boldsymbol{\zeta}_k) = 0, \ \hat{\boldsymbol{e}}_p \cdot \boldsymbol{\zeta}_k = 0, \ p \neq k, \qquad \boldsymbol{y} \in \partial \boldsymbol{y}_k, \tag{S1.6d}$$

$$\frac{\partial}{\partial y_j} \omega_k^a = 0, \ \frac{\partial}{\partial y_p} (\hat{\boldsymbol{e}}_p \cdot \boldsymbol{\zeta}_k) = 0, \ \hat{\boldsymbol{e}}_j \cdot \boldsymbol{\zeta}_k = 0, \ p \neq j, \ j \neq k, \qquad \boldsymbol{y} \in \partial \boldsymbol{y}_j, \tag{S1.6e}$$

where $\Omega_a^{1/8}$ is the $1/8$ th volume air domain, $\Gamma_{am}^{1/8}$ and $\Gamma_{as}^{1/8}$ are the $1/8$ th area solid and mixed phase boundaries, $\partial \boldsymbol{y}_k$ is the union of the boundaries at $y_k = 0$ and $y_k = 1/2$. The boundary

conditions (S1.6*d*) and (S1.6*e*) enforce a normal flow condition in the direction of forcing and a slip condition in the non-forced directions. The symmetry reduction is illustrated in Figure S1.1. In order to increase the clarity of the symmetry reduction we have illustrated this for an idealized sample in two dimensions. The boundary conditions (S1.6) are also applicable to a three dimensional soil image.

Similarly the symmetric form of the cell problem for the impeded Darcy flow of water in the mixed phase is

$$\nabla_y^2 \omega_k^m = 0, \qquad \boldsymbol{y} \in \Omega_m^{1/8}, \qquad \text{(S1.7}a\text{)}$$

$$\hat{\boldsymbol{n}}^{ms} \cdot \boldsymbol{\nabla}_y \omega_k^m + \hat{\boldsymbol{n}}^{ms} \cdot \hat{\boldsymbol{e}}_k = 0, \qquad \boldsymbol{y} \in \Gamma_{ms}^{1/8}, \qquad \text{(S1.7}b\text{)}$$

$$\hat{\boldsymbol{n}}^{am} \cdot \boldsymbol{\nabla}_y \omega_k^m + \hat{\boldsymbol{n}}^{am} \cdot \hat{\boldsymbol{e}}_k = 0, \qquad \boldsymbol{y} \in \Gamma_{am}^{1/8}, \qquad \text{(S1.7}c\text{)}$$

with the symmetry boundary conditions

$$\omega_k^m = 0, \qquad \boldsymbol{y} \in \partial \boldsymbol{y}_k, \qquad \text{(S1.7}d\text{)}$$

$$\frac{\partial}{\partial y_j} \omega_k^m = 0, \qquad \boldsymbol{y} \in \partial \boldsymbol{y}_j, \qquad \text{(S1.7}e\text{)}$$

where ω_k^m is the local pressure coefficient driven by a large scale pressure gradient in the mixed phase.

The symmetry boundary conditions for the cell problems, arising from the compressibility of the mixed phase, are more complex. We consider first the effect of strain on the soil displacement. From a macroscopic point of view, applying a strain to the soil sample will cause deformation of all components in the soil. However, as we have assumed that the soil minerals move as rigid bodies, they cannot be deformed. Hence, any macro-scale deformation must be coupled with a micro-scale variation in soil deformation which is equal and opposite to the macro-scale deformation on the solid grains (see [5] for a more formal derivation). This deformation is captured by imposing that on the solid particles the response to deformation can be calculated by solving

$$\boldsymbol{\nabla}_y \cdot \alpha_{pq}^u = 0, \qquad \boldsymbol{y} \in \Omega_m^{1/8}, \qquad \text{(S1.8}a\text{)}$$

$$\alpha_{pq} = e_y(\boldsymbol{\kappa}_{pq}^u) + \frac{\nu}{1-2\nu} \left(\boldsymbol{\nabla}_y \cdot \boldsymbol{\kappa}_{pq}^u \right) I, \qquad \boldsymbol{y} \in \Omega_m^{1/8}, \qquad \text{(S1.8}b\text{)}$$

$$\hat{\boldsymbol{n}}^{am} \cdot \left[\alpha_{pq}^u + \frac{\hat{\boldsymbol{e}}_p \hat{\boldsymbol{e}}_q + \hat{\boldsymbol{e}}_q \hat{\boldsymbol{e}}_p}{2} + \frac{\hat{\boldsymbol{e}}_p \cdot \hat{\boldsymbol{e}}_q}{2} \frac{\nu}{1-2\nu} I \right] = 0, \qquad \boldsymbol{y} \in \Gamma_{am}^{1/8}, \qquad \text{(S1.8}c\text{)}$$

where $\boldsymbol{\kappa}_{pq}^u$ is the displacement coefficient driven by a large scale strain gradient and α_{pq}^u is the corresponding stress coefficient. The boundary conditions on the boundary ∂y_k can be written in the compact form

$$\hat{\boldsymbol{e}}_k \cdot \left[\hat{\boldsymbol{e}}_p \hat{\boldsymbol{e}}_q + \hat{\boldsymbol{e}}_q \hat{\boldsymbol{e}}_p + \hat{\boldsymbol{e}}_p \hat{\boldsymbol{e}}_p + \hat{\boldsymbol{e}}_q \hat{\boldsymbol{e}}_q - I \right] \cdot \boldsymbol{\kappa}_{pq}^u = 0, \qquad \boldsymbol{y} \in \partial \boldsymbol{y}_k, \qquad \text{(S1.8}d\text{)}$$

$$\hat{\boldsymbol{e}}_k \cdot \left[\hat{\boldsymbol{e}}_p \hat{\boldsymbol{e}}_p + \hat{\boldsymbol{e}}_q \hat{\boldsymbol{e}}_q \right] \alpha_{pq}^u \left[\hat{\boldsymbol{e}}_p \hat{\boldsymbol{e}}_p + \hat{\boldsymbol{e}}_q \hat{\boldsymbol{e}}_q \right] \cdot \hat{\boldsymbol{e}}_k = 0, \; p \neq q, \qquad \boldsymbol{y} \in \partial \boldsymbol{y}_k, \qquad \text{(S1.8}e\text{)}$$

or

$$\hat{\boldsymbol{e}}_j \cdot \alpha_{pq}^u \cdot \hat{\boldsymbol{e}}_j = 0, \; j \neq k, \; p = q, \qquad \boldsymbol{y} \in \partial \boldsymbol{y}_k. \qquad \text{(S1.8}f\text{)}$$

These boundary conditions are illustrated for the case $p \neq q$ in Figure 3. The boundary conditions on the solid grains are more complex and we consider the cases $p = q$ and $p \neq q$ separately. For the case $p = q$ we observe that, for any solid grain that intersects a boundary, $y_k = 0$ or $y_k = 0.5$ for $k = \{1, 2, 3\}$, the net displacement of that grain in the direction $\hat{\boldsymbol{e}}_k$ must be zero. In addition we find that the stress condition projected into the direction $\hat{\boldsymbol{e}}_k$ is automatically satisfied. Therefore, for a particle that intersects the boundary $y_k = 0$ or $y_k = 0.5$ we have

$$\left[\boldsymbol{\kappa}^u_{pp} + (y_p - y_k)\hat{\boldsymbol{e}}_p\right] \cdot \hat{\boldsymbol{e}}_k = 0, \qquad \boldsymbol{y} \in \Gamma^{1/8}_{ms}. \tag{S1.8g}$$

Alternatively, if the particle does not intersect the boundary $y_k = 0$ or $y_k = 0.5$ then we apply the conditions

$$\left[\boldsymbol{\kappa}^u_{pp} + y_p\hat{\boldsymbol{e}}_p - \hat{\boldsymbol{\kappa}}_{pp}\right] \cdot \hat{\boldsymbol{e}}_k = 0, \qquad \boldsymbol{y} \in \Gamma^{1/8}_{ms}, \tag{S1.8h}$$

$$\int_{\Gamma^{1/8}_{ms,j}} \hat{\boldsymbol{n}}^{ms}_0 \cdot \alpha^u_{pp} \cdot \hat{\boldsymbol{e}}_k \, dy = 0, \tag{S1.8i}$$

where $\hat{\boldsymbol{\kappa}}_{pp}$ is an arbitrary constant. Similarly for the case $p \neq q$, if the solid grains intersect with $y_k = 0$ or $y_k = 0.5$ we apply

$$\left[\boldsymbol{\kappa}^u_{pq} + \frac{y_p\hat{\boldsymbol{e}}_q + y_q\hat{\boldsymbol{e}}_p}{2} - \hat{\boldsymbol{\kappa}}_{pq}\right] \cdot \hat{\boldsymbol{e}}_k = 0, \qquad \boldsymbol{y} \in \Gamma^{1/8}_{ms}, \tag{S1.8j}$$

$$\int_{\Gamma^{1/8}_{ms,j}} \hat{\boldsymbol{n}}^{ms} \cdot \alpha^u_{pq} \cdot \hat{\boldsymbol{e}}_k \, dy = 0, \tag{S1.8k}$$

and

$$\left[\boldsymbol{\kappa}^u_{pq} + \frac{y_p\hat{\boldsymbol{e}}_q + y_q\hat{\boldsymbol{e}}_p}{2}\right] \cdot \hat{\boldsymbol{e}}_j = 0, \; j \neq k, \qquad \boldsymbol{y} \in \partial\Gamma^{1/8}_{ms}, \tag{S1.8l}$$

where $\hat{\boldsymbol{\kappa}}_{pq}$ is an arbitrary constant. It is possible that a single particle can intersect multiple coordinate boundaries and could have zero motion in the y_1, y_2 and y_3 directions. Alternatively it may have zero motion in one of these directions but not others. Hence, considerable care must be taken in defining the location of the grain boundaries in relation to the domain boundaries, Figure 3.

The final cell problem describes the local deformation due to changes in pressure that move the soil away from the capillary static equilibrium, *i.e.*, $p^a_0 - p^c - p^m_0 \neq 0$. We write this as

$$\boldsymbol{\nabla}_y \cdot \alpha^p = 0, \qquad \boldsymbol{y} \in \Omega^{1/8}_m, \tag{S1.9a}$$

$$\alpha^p = e_y(\boldsymbol{\kappa}^p) + \frac{\nu}{1 - 2\nu}\left(\boldsymbol{\nabla}_y \cdot \boldsymbol{\kappa}^p\right) I, \qquad \boldsymbol{y} \in \Omega^{1/8}_m, \tag{S1.9b}$$

$$\hat{\boldsymbol{n}}^{am} \cdot \alpha^p = \hat{\boldsymbol{n}}^{am}, \qquad \boldsymbol{y} \in \Gamma_{am}, \tag{S1.9c}$$

where $\boldsymbol{\kappa}^p$ is the displacement coefficient driven by a the absolute pressure. The symmetry boundary conditions are

$$\hat{\boldsymbol{e}}_k \cdot \boldsymbol{\kappa}^p = 0, \qquad \boldsymbol{y} \in \partial y_k, \tag{S1.9d}$$

$$\hat{\boldsymbol{e}}_j \cdot \alpha^p \hat{\boldsymbol{e}}_j = 0, \; j \neq k, \qquad \boldsymbol{y} \in \partial y_k. \tag{S1.9e}$$

For a particle that intersects the boundary $y_k = 0$ or $y_k = 0.5$ we have

$$\boldsymbol{\kappa}^p \cdot \hat{\boldsymbol{e}}_k = 0, \qquad \boldsymbol{y} \in \Gamma^{1/8}_{ms}. \tag{S1.9f}$$

Alternatively, if the particle does not intersect the boundary $y_k = 0$ or $y_k = 0.5$ then we apply the conditions

$$\left[\boldsymbol{\kappa}^p - \hat{\boldsymbol{\kappa}}^{p,j}\right] \cdot \hat{\boldsymbol{e}}_k = 0, \qquad \boldsymbol{y} \in \Gamma_{ms}^{1/8}, \tag{S1.9g}$$

$$\int_{\Gamma_{ms,j}^{1/8}} \hat{\boldsymbol{n}}^{ms} \cdot \alpha^p \cdot \hat{\boldsymbol{e}}_k \, dy = 0, \tag{S1.9h}$$

where $\hat{\boldsymbol{\kappa}}^p$ is a constant vector.

Equations (S1.6), (S1.7), (S1.8) and (S1.9) are the cell problems presented in the main body of the paper, *i.e.*, equations (3.2), (3.3), (3.4) and (3.5).

(c) The averaged model

Using the results from the cell problems the final stage of the homogenisation procedure constitutes a volume average [5]. This approach was used to obtain a set of macroscopic equations for the solid displacement $\boldsymbol{u}^m$, the air pressure p^a and the fluid pressure in the mixed phase p^m, parametrised by the underlying geometry. In the original paper [5], this final stage of averaging resulted in 20 effective tensor quantities. Due to the symmetry of the cell problems in this study, several of these parameters become identically zero. Hence, we present the final dimensionless equations for the symmetric case. For the elastic displacement of the solid the averaged force balance is

$$\begin{aligned}\frac{\partial}{\partial x_i}\Bigg\{ & C^u_{ijkl}\left[\frac{\partial u^m_{0k}}{\partial x_l} + \frac{\partial u^m_{0l}}{\partial x_k}\right] + C^p_{ij}(p^w_0 + p^c - p^a_0) \\ & - \left(\delta_{ij} - \frac{1}{||\mathcal{B}_m||}\int_{\partial\mathfrak{B}_{ms}} \hat{n}^{ms}_{0j} y_i \, dy\right) p^w_0 + \left(\frac{||\mathcal{B}_a||}{||\mathcal{B}_m||}\delta_{ij} - \frac{1}{||\mathcal{B}_m||}\int_{\partial\mathfrak{B}_{as}} \hat{n}^{as}_{0j} y_i \, dy\right) p^a_0\Bigg\} = g^{eff}\hat{\boldsymbol{e}}_3,\end{aligned} \tag{S1.10}$$

where C^u_{ijkl} is the stiffness tensor and C^p_{ij} is the pressure induced stress coefficient respectively, these are given by

$$\begin{aligned} C^u_{ijkl} = & \frac{1}{||\mathcal{B}_m||}\int_{\mathfrak{B}_m}\left[\frac{\partial \kappa^u_{kli}}{\partial y_j} + \frac{\partial \kappa^u_{klj}}{\partial y_i} + \delta_{ik}\delta_{jl} + \frac{\nu}{1-2\nu}\left(\delta_{ij}\delta_{kl} + \boldsymbol{\nabla}_y \cdot \boldsymbol{\kappa}^u_{kl}\delta_{ij}\right)\right] d\boldsymbol{y} \\ - & \frac{1}{||\mathcal{B}_m||}\int_{\partial\mathfrak{B}_{ms}} y_i \hat{n}^{ms}_{0q}\left[\frac{\partial \kappa^u_{klq}}{\partial y_j} + \frac{\partial \kappa^u_{klj}}{\partial y_q} + \delta_{qk}\delta_{jl} + \frac{\nu}{1-2\nu}\left(\delta_{qj}\delta_{kl} + \boldsymbol{\nabla}_y \cdot \boldsymbol{\kappa}^u_{kl}\delta_{qj}\right)\right] d\boldsymbol{y}, \end{aligned} \tag{S1.11a}$$

$$\begin{aligned} C^p_{ij} = & \frac{1}{||\mathcal{B}_m||}\int_{\mathfrak{B}_m}\left[\frac{\partial \kappa^p_i}{\partial y_j} + \frac{\partial \kappa^p_j}{\partial y_i} + \frac{\nu}{1-2\nu}\boldsymbol{\nabla}_y \cdot \boldsymbol{\kappa}^p \delta_{ij}\right] d\boldsymbol{y} \\ - & \frac{1}{||\mathcal{B}_m||}\int_{\partial\mathfrak{B}_{ms}} y_i \hat{n}^{ms}_{0q}\left[\frac{\partial \kappa^p_q}{\partial y_j} + \frac{\partial \kappa^p_j}{\partial y_q} + \frac{\nu}{1-2\nu}\boldsymbol{\nabla}_y \cdot \boldsymbol{\kappa}^p \delta_{qj}\right] d\boldsymbol{y}, \end{aligned} \tag{S1.11b}$$

and

$$g^{eff} = g^w\left\{[\phi + \delta_2(1-\phi)] + \frac{||\mathfrak{B}_a||}{||\mathfrak{B}_m||}\delta_3 + \frac{||\mathfrak{B}_s||}{||\mathfrak{B}_m||}\delta_4\right\}. \tag{S1.11c}$$

The equation for the fluid pressure in the mixed phase is

$$\frac{\partial}{\partial t}\left\{\frac{\partial u^m_j}{\partial x_j} + A^u_{ij}\left[\frac{\partial u^m_i}{\partial x_j} + \frac{\partial u^m_j}{\partial x_i}\right] + A^p\left(p^c + p^m - p^a\right)\right\} = \frac{\partial}{\partial x_j}\left[K^w_{jk}\left(\frac{\partial p^m}{\partial x_k} + g^w\delta_{k3}\right)\right], \tag{S1.12}$$

where K^w_{ij} is the effective water mobility, A^u_{ij} and A^p are the increment of water content due to strain and pressure changes respectively. They are given by

$$K^w_{ij} = \frac{1}{||\mathfrak{B}_m||}\int_{\mathfrak{B}_m} \delta_{ij} + \frac{\partial \omega^m_j}{\partial y_i} \, d\boldsymbol{y}, \tag{S1.13a}$$

$$A_{ij}^{u}=\frac{1}{||\mathfrak{B}_m||}\int_{\mathfrak{B}_m}\boldsymbol{\nabla}_y\cdot\boldsymbol{\kappa}_{ij}^{u}\,d\boldsymbol{y}, \tag{S1.13b}$$

$$A^{p}=\frac{1}{||\mathfrak{B}_m||}\int_{\mathfrak{B}_m}\boldsymbol{\nabla}_y\cdot\boldsymbol{\kappa}^{p}\,d\boldsymbol{y}. \tag{S1.13c}$$

Finally the equation for the air pressure is

$$\begin{aligned}&\frac{\partial}{\partial t}\left\{\frac{||\mathfrak{B}_a||}{||\mathfrak{B}_m||}\frac{\partial u_j^m}{\partial x_j}+B_{ij}^{u}\left[\frac{\partial u_i^m}{\partial x_j}+\frac{\partial u_j^m}{\partial x_i}\right]+B^{p}\left(p^c+p^m-p^a\right)\right\}\\&=\frac{\partial}{\partial x_i}\left[K_{ij}^{a}\left(\frac{\partial p^a}{\partial x_j}+g^{w}\delta_3\delta_{k3}\right)\right],\end{aligned} \tag{S1.14}$$

where K_{ij}^{a} is the effective air mobility, B_{ij}^{u} and B^{p} are the increment of air content due to strain and pressure changes respectively. They are given by

$$K^{a}=-\frac{1}{\delta_1||\mathfrak{B}_a||}\int_{\mathfrak{B}_a}\boldsymbol{\zeta}_k\otimes\hat{\boldsymbol{e}}_k\,d\boldsymbol{y}, \tag{S1.15a}$$

$$B_{ij}^{u}=\frac{1}{||\mathfrak{B}_a||}\int_{\partial\mathfrak{B}_{am}}\hat{\boldsymbol{n}}^{am}\cdot\boldsymbol{\kappa}_{ij}^{u}\,d\boldsymbol{y}+\frac{1}{||\mathfrak{B}_a||}\sum_j\int_{\partial\mathfrak{B}_{as,j}}\hat{\boldsymbol{n}}^{am}\cdot\boldsymbol{\gamma}_{ij}^{u}\,d\boldsymbol{y}, \tag{S1.15b}$$

$$B^{p}=\frac{1}{||\mathfrak{B}_a||}\int_{\partial\mathfrak{B}_{am}}\hat{\boldsymbol{n}}^{am}\cdot\boldsymbol{\kappa}^{p}\,d\boldsymbol{y}+\frac{1}{||\mathfrak{B}_a||}\sum_j\int_{\partial\mathfrak{B}_{as,j}}\hat{\boldsymbol{n}}^{am}\cdot\boldsymbol{\gamma}^{p}\,d\boldsymbol{y}. \tag{S1.15c}$$

These equations provide a complete description of how the micro-scale geometry and mechanical properties influences the macro-scale mechanical properties of the poro-elastic composite. We note that if we consider the capillary static limit with an incompressible mixed phase then $A_{ij}^{u}=A^{u}\delta_{ij}$ and $B_{ij}^{u}=B^{u}\delta_{ij}$, and equations (S1.10) and (S1.14) reduce to Darcy's law for the water and air phases respectively.

The parameters given in equations (S1.13), (**??**) and (S1.15) are the equations given in the main body of the paper, *i.e.*, equations (3.8), (3.6) and (3.9).

At this point we consider the effect of solving equations (S1.6) on a $1/8$-th geometry. We notice that for the k-th cell problem, as $\hat{\boldsymbol{e}}_k\cdot\boldsymbol{\zeta}_k$ is even on all boundaries, we would need to multiply the result by a factor of 8 to account for the $7/8$ths of the geometry not considered. However, as the effective parameters are all normalized by the cell volume, this factor of 8 will cancel out. In addition, as each of the remaining velocity components is odd about two boundaries, we find

$$\int_{\Omega_a}\hat{\boldsymbol{e}}_j\cdot\boldsymbol{\zeta}_k\,d\boldsymbol{y}=0,\ j\neq k. \tag{S1.16}$$

Hence, K_{ij}^{a} is zero for $p\neq q$. By a similar analysis as for (S1.6) we find that K_{ij}^{w}, C_{ij}^{p}, A_{ij}^{u} and B_{ij}^{u} are zero if $i\neq j$, and C_{ijkl}^{u} is zero if $i\neq k$ and $j\neq l$. In summary, the effective equations describe a three phase poroelastic material and are parameterised by 9 effective parameters. These are; g^{eff} the effective gravitational force; C_{ijkl}^{u}, the macro-scale or effective stiffness tensor; C_{ij}^{p} the stress coefficient for pressure variations about the capillary static point; K_{ij}^{w} and K_{ij}^{a}, the water and air relative mobilities; and A_{ij}^{u}, A^{p}, B_{ij}^{u} and B^{p}, the strain and pressure induced variations in water and air content.

References

1. Maurice A Biot.
   General theory of three-dimensional consolidation.
   Journal of applied physics, 12(2):155–164, 1941.
2. MA Biot and DG Willis.
   The elastic coeff cients of the theory of consolidation.
   J. appl. Mech, 24:594–601, 1957.
3. Maurice A Biot.
   Theory of elasticity and consolidation for a porous anisotropic solid.

Journal of applied physics, 26(2):182–185, 1955.

4. AC Fowler and CG Noon.
Mathematical models of compaction, consolidation and regional groundwater flow.
Geophysical Journal International, 136(1):251–260, 1999.
5. Keith R Daly and Tiina Roose.
Determination of macro-scale soil properties from x-ray computed tomography - model derivation.
Submitted to: Proceedings of The Royal Society A: Mathematical Physical and Engineering Sciences, 2017.
6. G.S. Beavers and D.D. Joseph.
Boundary conditions at a naturally permeable wall.
J. Fluid Mech, 30(1):197–207, 1967.
7. S.R. Tracy, K. R. Daly, C.J. Sturrock, N.M.J Crout, S.J. Mooney, and T. Roose.
Three dimensional quantification of soil hydraulic properties using x-ray computed tomography and image based modelling.
Submitted to WRR, 2014.
8. Jacob Bear and Yehuda Bachmat.
Introduction to modeling of transport phenomena in porous media, volume 4.
Springer Science & Business Media, 2012.
