# Supplementary material for: Determination of macro-scale soil properties from pore scale structures: image-based modelling of poroelastic structures
Source: Proc Math Phys Eng Sci. 2018 Jul 11;474(2215):20170745. doi: 10.1098/rspa.2017.0745 (PMC6083241; doi:10.1098/rspa.2017.0745)
Supplement: Supplementary 02 [file rspa20170745supp2.pdf]

S2. Additional plots

In this supplementary section we provide additional plots for the parameters $||\Omega^i||$ for $i = \{a, m, s\}$, K^a_{ij} and C^p_{ij}, the discussion relating to these plots is given in the main body of the paper.

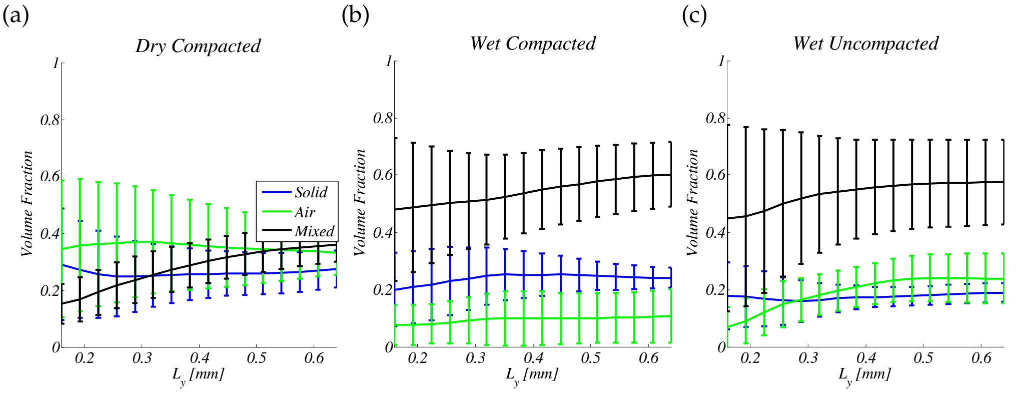

Figure S2.1. The volume fractions of the different soil phases for each soil treatment. Showing (a) the dry compacted phase (minimum 5 replicates, maximum 5 replicates), (b) the wet compacted phase (minimum 4 replicates), and (c) the wet uncompacted phase (minimum 3 replicates). The error bars show the standard deviation across the converged replicates

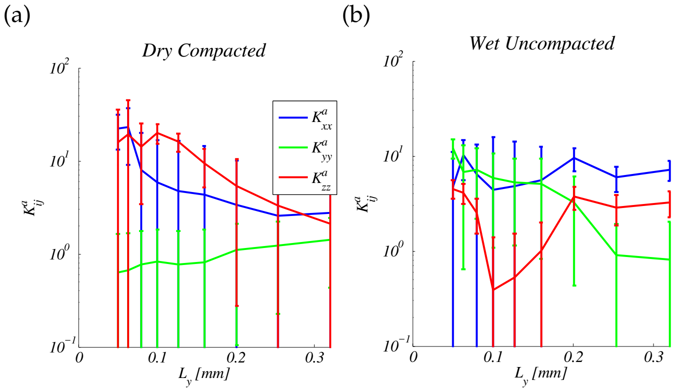

Figure S2.2. Air mobility (K^a_{ij} for $i = j$) based on $k = 10^{-10}$ m^2, $\mu^w = 8.9 \times 10^{-4}$ Pa s and $\mu^a = 1.8 \times 10^{-5}$ Pa s. Showing (a) the dry compacted phase (2 replicates in all cases), and (b) the wet uncompacted phase (2 replicates in all cases). We note the air mobility of the wet compacted treatment is zero, hence, the results are not shown. The error bars show the standard deviation across the converged replicates

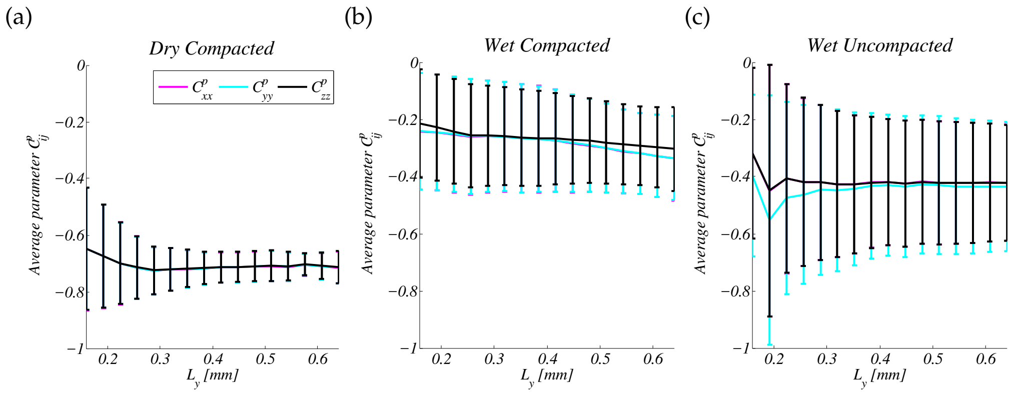

Figure S2.3. Effective parameter describing the change in stress due to a change in fluid pressure in the mixed phase (C^p_{ij} for $i = j$). Showing (a) the dry compacted phase (minimum 6 replicates), (b) the wet compacted phase (minimum 6 replicates), and (c) the wet uncompacted phase (minimum 5 replicates). The error bars show the standard deviation across the converged replicates
